# Modeling and Analysis of Unsteady Axisymmetric Squeezing Fluid Flow through Porous Medium Channel with Slip Boundary

**DOI:** 10.1371/journal.pone.0117368

**Published:** 2015-03-04

**Authors:** Mubashir Qayyum, Hamid Khan, M. Tariq Rahim, Inayat Ullah

**Affiliations:** Department of Mathematics, National University of Computer & Emerging Sciences - FAST Peshawar Campus, Peshawar, 25000, Pakistan; Massachusetts Institute Of Technology, UNITED STATES

## Abstract

The aim of this article is to model and analyze an unsteady axisymmetric flow of non-conducting, Newtonian fluid squeezed between two circular plates passing through porous medium channel with slip boundary condition. A single fourth order nonlinear ordinary differential equation is obtained using similarity transformation. The resulting boundary value problem is solved using Homotopy Perturbation Method (HPM) and fourth order Explicit Runge Kutta Method (RK4). Convergence of HPM solution is verified by obtaining various order approximate solutions along with absolute residuals. Validity of HPM solution is confirmed by comparing analytical and numerical solutions. Furthermore, the effects of various dimensionless parameters on the longitudinal and normal velocity profiles are studied graphically.

## Introduction

The interest in behavior of fluid flow through porous media began in the early days of oil and gas production, where the focus was on estimating and optimizing production. Similarly, another important application is the simulation of ground water pollution, mostly occurring due to leakage of chemicals from tanks and oil pipelines. The objective is to consider groundwater as one medium and polluted water as another, so that the spreading in the latter medium and its consequences can be studied.

In recent times, after the introduction of the modified Darcy Law [1], analysis through porous medium has been an important topic for the research community, as it finds is use in fields such as reservoir, petroleum, chemical, civil, environmental, agricultural, and biomedical engineering. Some practical applications in these fields include chemical reactors, filtration, geothermal reservoirs, ground water hydrology, drainage and recovery of crude oil from pores of reservoir rocks [2–7].

Squeezing flow has attracted significant attention because of its broad applications in many fields such as chemical, mechanical, and industrial engineering, and in bio-mechanics and food industries. Practical applications of squeezing flows in these fields are polymer processing, modeling of lubrication systems, and compression and injection molding, etc. These flows are induced by applying normal stresses or vertical velocities by means of a moving boundary, which can be frequently observed in various hydro-dynamical tools and machines.

Pioneering work on squeezing flows was investigated by Stefan [8] in which he proposed an adhoc asymptotic solution of Newtonian fluid. A solution considering inertial terms was found by Thorp [9]. However, Gupta and Gupta [10] later showed that this solution failed to satisfy boundary conditions. The effect of the inertial term in squeezing films between circular plates has been evaluated by Kuzma [11]. Elkouh [12] studied the squeeze film between two plane annuli taking fluid inertia effects under consideration. Verma [13] and Singh et al. [14] set up numerical solutions of the squeezing flows between parallel plates. Leider and Bird [15] carried out theoretical analysis for squeezing flow of power-law fluid between parallel plates. Naduvinamani et al. [16] investigated squeeze film lubrication of a short porous journal with couple stress fluids. Steady axisymmetric squeezing fluid flow in a porous medium has been analyzed by Islam et al. [17]. Hamza [18] worked on squeeze films considering MHD effect. Suction and injection effects on the flow of electrically conducting viscous fluid squeezed between two parallel disks was studied by Domairry et al. [19]. The study of the porosity and squeezing effects, while investigating the unsteady squeezing flow of visco-elastic Jeffery fluid between parallel disks, has been performed by Qayyum et al. [20]. Apart from the mentioned scholars, other researchers have also carried out different theoretical and experimental studies of squeezing flows [21–24].

No-slip boundary condition is one of the main concepts of fluid dynamics. Consider a liquid flowing over a solid wall. The condition in which the liquid molecules near the solid wall are motionless, relative to the wall, is called no-slip boundary [25]. This boundary condition has been employed in modeling various viscous and visco-elastic fluid flow problems. Firstly, Navier [26] proposed the general boundary condition which shows fluid slip at the liquid-solid interface. According to him, the difference between the boundary and fluid velocities is proportional to the shear stress at the boundary. The dimension of proportionality constant is length, and this is known as the slip parameter. There are numerous situations in which no-slip boundary condition is not appropriate. For instance, flow on multiple interfaces, polymeric liquids when the weight of the molecules is high, fluids containing concerted suspensions, and thin film problems.

A number of perturbation techniques which can solve non-linear boundary value problems analytically are discussed in literature. But the assumption of small parameter is a limitation in these techniques. Recently, a technique was proposed by He [27–30], that combines homotopy and the traditional perturbation method [31– 34]. This technique was the beginning of homotopy perturbation method (HPM). In a series of papers, He applied this method to discuss non-linear boundary value problems [27–30]. As a result, many researchers have used HPM to solve non-linear differential equations in different fields as it is not only easy to use, but also successful. This method minimizes the limitations commonly associated with perturbation techniques, while taking full advantage of the traditional perturbation methods. In fluid dynamics, Siddiqui et al. [35, 36] applied this technique for solving non-linear boundary value problems arising in Newtonian and non-Newtonian fluids. In addition, Zhou and Wu [37] used this technique in an inverse heat problem. Also, Hamid et al. [38] compared the method with other analytical and numerical techniques, while solving higher order non-linear differential equations.

The objective of this manuscript is to use HPM for the solution of an unsteady axisymmetric squeezing fluid flow between two circular plates through porous medium with slip boundary condition. Validity of HPM solution is confirmed by comparing analytical and numerical solutions. In addition, effects of different dimensionless parameters on the velocity profiles are studied graphically.

## Description of the Problem

**Fig 1:**
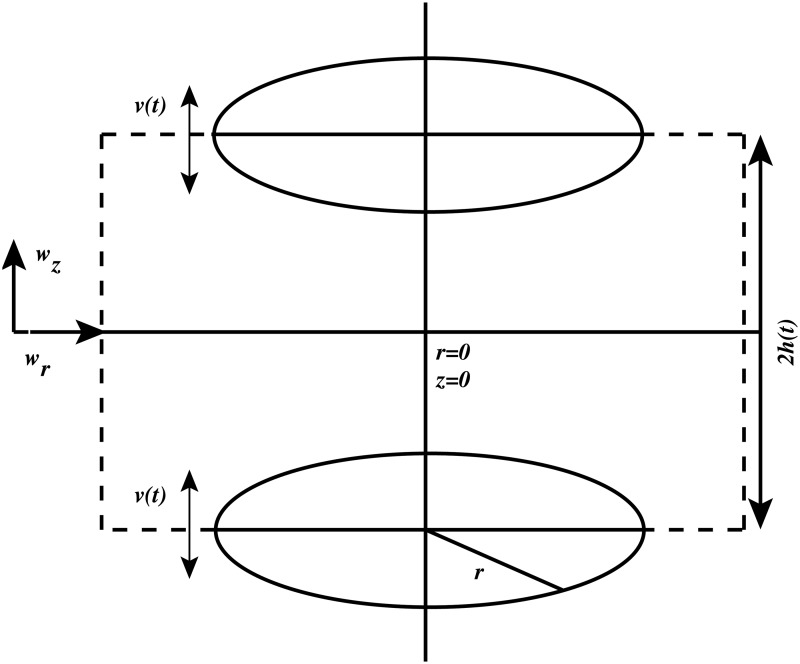
Geometry of the flow. Squeezing flow between two circular plates having distance 2*h*(*t*). *r*-axis is the central axis of the channel while *z*-axis is taken normal to it. The plates move symmetrically with respect to *z* = 0 at a speed *v*(*t*) while the flow is axisymmetric about *r* = 0. Longitudinal and normal velocity components in radial and axial directions are *w*_*r*_(*r*,*z*,*t*) and *w*_*z*_(*r*,*z*,*t*) respectively.

An unsteady axisymmetric squeezing flow of incompressible first grade fluid with density *ρ*, viscosity *μ*, and kinematic viscosityυ, squeezed between two circular plates having speed *v*(*t*) and passing through porous medium channel is considered. It is assumed that at any time *t*, the distance between the two circular plates is 2*h*(*t*). Also, it is assumed that *r*-axis is the central axis of the channel while *z*-axis is taken normal to it. Plates move symmetrically with respect to the central axis *z* = 0 while the flow is axisymmetric about *r* = 0. The longitudinal and normal velocity components in radial and axial directions are *w*_*r*_(*r*,*z*,*t*) and *w*_*z*_(*r*,*z*,*t*) respectively. The geometrical representation of the flow is illustrated in Fig. 1.

## Problem Formulation

The basic governing equations of motion are

∇⋅W=0 (1)

$$\rho [\frac{\partial W}{\partial t}+(W\cdot \nabla )W]=\rho \text{f}\;+\nabla \cdot T+\tilde{r}$$ (2)

where

T=-pI+μA (3)

$$A=\nabla \;W+{\left(\nabla \;W\right)}^{t}$$ (4)

and **W** is the velocity vector, *p* is the pressure, f is the body force, **T** is the Cauchy stress tensor, **A** is the Rivlin-Ericksen tensor, μ is the coefficient of viscosity, and $\tilde{r}$ is the Darcy’s resistance. According to Breugem equation [39], $\tilde{r}$can be written as:

$$\tilde{r}=-\frac{\mu }{k}\;W$$ (5)

where *k* is the permeability constant.

Now, we formulate the unsteady two-dimensional flow through porous medium. After neglecting body force we assume that

$$W=[w_{r}(r,z,t),0,w_{z}(r,z,t)]$$ (6)

and introduce the vorticity function Ω (*r*,*z*,*t*)and generalized pressure $\overset{⌢}{P}(r,z,t)$as

$$\Omega (r,z,t)=\frac{\partial w_{z}}{\partial r}-\frac{\partial w_{r}}{\partial z}$$ (7)

$$\overset{⌢}{P}(r,z,t)=\frac{\rho }{2}[w_{r}^{2}+w_{z}^{2}]+p$$ (8)

Equations (1) and (2) can then be reduced to

$$\frac{\partial w_{r}}{\partial r}+\frac{w_{r}}{r}+\frac{\partial w_{z}}{\partial z}=0$$ (9)

$$\frac{\partial \overset{⌢}{P}}{\partial r}+\rho (\frac{\partial w_{r}}{\partial t}-w_{z}\Omega )=-\mu (\frac{\partial \Omega }{\partial z}+\frac{w_{r}}{k})$$ (10)

$$\frac{\partial \overset{⌢}{P}}{\partial z}+\rho (\frac{\partial w_{z}}{\partial t}+w_{r}\Omega )=\mu (\frac{1}{r}\frac{\partial }{\partial r}(r\Omega )-\frac{w_{z}}{k})$$ (11)

The boundary conditions on *w*_*r*_(*r*,*z*,*t*) and *w*_*z*_(*r*,*z*,*t*) are

wr(r,z,t)=β∂∂zwr(r,z,t)andwz(r,z,t)=v(t)atz=h∂∂zwr(r,z,t)=0andwz(r,z,t)=0atz=0 (12)

where $v(t)=\frac{dh}{dt}$ is the velocity of the plates. The boundary conditions in (12) are due to slip at the upper plate when z = h and symmetry at z = 0. If we launch the dimensionless parameter

$$\xi =\frac{z}{h(t)}$$ (13)

Equations (7),(9),(10)and(11)are converted to

$$\Omega (r,z,t)=\frac{\partial w_{z}}{\partial r}-\frac{1}{h}\frac{\partial w_{r}}{\partial \xi }$$ (14)

$$\frac{\partial w_{r}}{\partial r}+\frac{w_{r}}{r}+\frac{1}{h}\frac{\partial w_{z}}{\partial \xi }=0$$ (15)

$$\frac{\partial \overset{⌢}{P}}{\partial r}+\rho (\frac{\partial w_{r}}{\partial t}-w_{z}\Omega )=-\mu (\frac{1}{h}\frac{\partial \Omega }{\partial \xi }+\frac{w_{r}}{k})$$ (16)

$$\frac{1}{h}\frac{\partial \overset{⌢}{P}}{\partial \xi }+\rho (\frac{\partial w_{z}}{\partial t}+w_{r}\Omega )=\mu (\frac{1}{r}\frac{\partial }{\partial r}(r\Omega )-\frac{w_{z}}{k})$$ (17)

The boundary conditions on *w*_r_ and *w*_z_ are

wr=β1h∂wr∂ξandwz=v(t)atξ=1∂wr∂ξ=0andwz=0atξ=0. (18)

After eliminating the $\overset{⌢}{P}(r,z,t)$ between (16) and (17), we obtain:

$$\rho [\frac{\partial \Omega }{\partial t}+w_{r}\frac{\partial \Omega }{\partial r}+\frac{w_{z}}{h}\frac{\partial \Omega }{\partial \xi }-\frac{w_{r}}{r}\Omega ]=\mu [\nabla^{2}\Omega -(\frac{1}{r^{2}}+\frac{1}{k})\Omega ]$$ (19)

where ▽^2^ is the Laplacian operator.

Defining velocity components as [11]

wr=-r2h(t)v(t)F’(ξ)wz=v(t)F(ξ) (20)

we see that (15) is identically satisfied and therefore, (19) becomes

$$\frac{d^{4}F}{d\xi^{4}}+R[(\xi -F)\frac{d^{3}F}{d\xi^{3}}+2\frac{d^{2}F}{d\xi^{2}}]-Q\frac{d^{2}F}{d\xi^{2}}-M\frac{d^{2}F}{d\xi^{2}}=0$$ (21)

where

$$R=\frac{h\;v(t)}{\upsilon },Q=\frac{h^{2}}{\upsilon v(t)}\frac{dv(t)}{dt}\;\text{and}\;M=\frac{h^{2}}{k}$$ (22)

Both *R* and *Q* are functions of time but for similarity solution we consider *R* and *Q* constants. Since$v=\frac{dh}{dt}$, integrating the first equation of (22), we obtain:

$$h(t)={\left(Ct+D\right)}^{\frac{1}{2}}$$ (23)

where *C* and *D* are constants. When *C*>0 and *D*>0, the plates move away from each other symmetrically with respect to ξ. The squeezing flow exists when the plates approach each other when *C*>0, *D*>0 and *h*(*t*)>0. From (22) and (23) it follows that *Q* = -*R*. Then (21) becomes

$$\frac{d^{4}F}{d\xi^{4}}+R[(\xi -F)\frac{d^{3}F}{d\xi^{3}}+3\frac{d^{2}F}{d\xi^{2}}]-M\frac{d^{2}F}{d\xi^{2}}=0$$ (24)

After using (18) and (20), we establish the following boundary conditions in case of slip at the upper plate:

F(1)=1,F’(1)=γF″(1)F(0)=0,F″(0)=0 (25)

## Fundamental Theory of HPM [27–30]

To exhibit the basic theory of HPM, let us consider the following differential equation:

L(w)+N(w)-g(r)=0, r∈ΩB(w,dwdn)=0, r∈ϒ (26)

where *w* is an unknown function and *g*(*r*) is a known function. *L*, *N*, *B* are linear, nonlinear and boundary operators respectively. Also ϒ is the boundry of the domain Ω.

We construct Homotopy θ(r,p):Ω×[0,1]→R which satisfies

$$\psi (\theta ,p)=(1-p)[L(\theta )-L(w_{0})]+p[L(\theta )+N(\theta )-g(r)]=0,\;r\in \Omega$$ (27)

where *p* ϵ [0, 1] is an embedding parameter, and *w*_0_ is the initial guess of (26) which satisfies the boundary conditions. From (27), we have:

ψ(θ,0)=L(θ)-L(w0)=0ψ(θ,1)=L(θ)+N(θ)-g(r)=0 (28)

Thus, as *p* varies from 0 to1, the solution *θ*(*r*,*p*) approaches from *w*_0_(r) to$\tilde{w}(r)$.

To obtain an approximate solution, we expand *θ*(*r*,*p*) in a Taylor series about *p* as follows: $\theta (r,p)=\theta_{0}+\sum_{k=1}^{\infty }\theta_{k}p^{k}$ Setting p = 1, the approximate solution of (26) would be

$$\tilde{w}=\underset{p\to 1}{lim}\theta (r,p)=\sum_{k=1}^{\infty }\theta_{k}$$ (29)

## Application of HPM

Using (24) and (25), various order problems are as follows: Zeroth-Order Problem

u0(iv)(ξ)=0,u0(0)=0,u″0(0)=0,u0(1)=1,u’0(1)=γu″0(1) (30)

First-Order Problem

u1(iv)(ξ)-Mu″0(ξ)+3Ru″0(ξ)+Rξu‴0(ξ)-Ru0(ξ)u‴0(ξ)=0,u1(0)=0,u″1(0)=0,u1(1)=0,u’1(1)=γu″1(1) (31)

Second-Order Problem

u2(iv)(ξ)-Mu″1(ξ)+3Ru″1(ξ)-Ru1(ξ)u‴0(ξ)+Rξu‴1(ξ)-Ru0(ξ)u‴1(ξ)=0,u2(0)=0,u″2(0)=0,u2(1)=0,u’2(1)=γu″2(1) (32)

Third-Order Problem

u3(iv)(ξ)-Mu″2(ξ)+3Ru″2(ξ)-Ru2(ξ)u‴0(ξ)-Ru1(ξ)u‴1(ξ)+Rξu‴2(ξ)-Ru0(ξ)u‴2(ξ)=0,u3(0)=0,u″3(0)=0,u3(1)=0,u’3(1)=γu″3(1) (33)

Fourth-Order Problem

u4(iv)(ξ)-Mu″3(ξ)+3Ru″3(ξ)-Ru3(ξ)u‴0(ξ)-Ru2(ξ)u‴1(ξ)-Ru1(ξ)u‴2(ξ)+Rξu‴3(ξ)-Ru0(ξ)u‴3(ξ)=0,u4(0)=0,u″4(0)=0,u4(1)=0,u’4(1)=γu″4(1) (34)

Fifth-Order Problem

u5(iv)(ξ)-Mu″4(ξ)+3Ru″4(ξ)-Ru4(ξ)u‴0(ξ)-Ru3(ξ)u‴1(ξ)-Ru2(ξ)u‴2(ξ)-Ru1(ξ)u‴3(ξ)+Rξu‴4(ξ)-Ru0(ξ)u‴4(ξ)=0,u5(0)=0,u″5(0)=0,u5(1)=0,u’5(1)=γu″5(1) (35)

By considering fifth order solution, we have

$$\tilde{u}(\xi )=\sum_{i=0}^{5}u_{i}(\xi )$$ (36)

Keeping *R* = 1, *M* = 1 and *γ*, the approximate solution is

u~(ξ)={0.634674ξ+0.409456ξ3-0.0482189ξ5+0.00465172ξ7-0.000635324ξ9+0.0000811794ξ11-9.86308×10-6ξ13+1.00747×10-6ξ15-8.04603×10-8ξ17+4.41816×10-9ξ19-1.36769×10-10ξ21+1.54292×10-12ξ23} (37)

The residual of the problem is

$$R=\frac{d^{4}\tilde{u}}{d\xi^{4}}+R[(\xi -\tilde{u})\frac{d^{3}\tilde{u}}{d\xi^{3}}+3\frac{d^{2}\tilde{u}}{d\xi^{2}}]-M\frac{d^{2}\tilde{u}}{d\xi^{2}}$$ (38)

If ℜ = 0, then $\tilde{u}$ will be the exact solution, but usually this does not occur in non-linear problems.

## Results and Discussions

In the present article, we considered an unsteady axisymmetric squeezing flow of incompressible Newtonian fluid passing through porous medium with slip boundary condition. The resulting non-linear boundary value problem is solved through HPM and RK4.

There are three parameters; Reynolds number *R*, constant containing permeability *M*, and slip parameter γ in the current problem. We present our discussion of results based on different compositions of these parameters. First of all we solve the problem for various values of R,Mandγanalytically using HPM. This is illustrated in Tables 1, 2, and 3. Secondly, we solve the problem numerically using RK4 for various *R*,*M* and *γ*. This is explained in Tables 4, 5, and 6. We also check the convergence of HPM solution using different order approximations in Table 7. Finally, we check the validity of HPM solutions by comparing analytical and numerical solutions. This is demonstrated in S1, S2 and S3 Table. All the tables signify the efficiency of HPM. Furthermore, we investigated the effects of various dimensionless parameters on the normal and longitudinal velocity profiles graphically.

**Table 1:** HPM solutions along with absolute residuals for various *R* when *γ* = 1 and *M* = 0.3.

ξ	*R* = 0.7		*R* = 0.9		*R* = 1.0	
	Solution	Residual	Solution	Residual	Solution	Residual
0.0	0.	0.	0.	0.	0.	0.
0.1	0.0779056	7.94082×10^-7^	0.0758376	1.07178×10^-9^	0.0746634	2.25321×10^-9^
0.2	0.157102	1.2091×10^-6^	0.15132	1.88369×10^-9^	0.150878	2.65694×10^-9^
0.3	0.23889	9.05355×10^-7^	0.23334	2.10531×10^-9^	.0230192	9.23882×10^-10^
0.4	0.32459	3.44432×10^-7^	0.317923	1.41494×10^-9^	0.314146	1.56557×10^-9^
0.5	0.415552	2.53517×10^-6^	0.408346	2.23822×10^-10^	0.404268	3.09459×10^-9^
0.6	0.513164	5.28935×10^-6^	0.50608	2.26552×10^-9^	0.502076	2.99595×10^-9^
0.7	0.618869	7.77624×10^-6^	0.612606	3.67602×10^-9^	0.609071	1.86451×10^-9^
0.8	0.734172	8.81226×10^-6^	0.729421	3.68216×10^-9^	0.726742	7.09482×10^-10^
0.9	0.860657	7.25419×10^-6^	0.858038	2.57384×10^-9^	0.856564	1.02005×10^-10^
1.0	1.	2.67891×10^-6^	1.	1.04787×10^-9^	1.	8.59397×10^-21^

**Table 2:** HPM solutions along with absolute residuals for various *M* when *γ* = 1 and *R* = 0.3.

ξ	*M* = 0.5		*M* = 0.7		*M* = 0.9	
	Solution	Residual	Solution	Solution	Residual	Solution
0.0	0.	0.	0.	0.	0.	0.
0.1	0.0734874	2.1267×10^-7^	0.0743047	9.40321×10^-9^	0.0750771	1.62039×10^-12^
0.2	0.148618	3.80716×10^-7^	0.150187	1.62781×10^-8^	0.151669	1.91338×10^-12^
0.3	0.227027	4.7353×10^-7^	0.229219	1.90699×10^-8^	0.231291	6.71317×10^-13^
0.4	0.310335	4.83136×10^-7^	0.312965	1.77119×10^-8^	0.315453	1.11729×10^-12^
0.5	0.400138	4.24408×10^-7^	0.402978	1.34725×10^-8^	0.405665	2.21927×10^-12^
0.6	0.498003	3.27592×10^-7^	0.500792	8.27910×10^-9^	0.503432	2.15310×10^-12^
0.7	0.605459	2.26980×10^-7^	0.607922	3.91263×10^-9^	0.610254	1.34291×10^-12^
0.8	0.723994	1.50704×10^-7^	0.72586	1.47900×10^-9^	0.727628	5.12541×10^-13^
0.9	0.855046	1.15451×10^-7^	0.856073	1.35555×10^-9^	0857047	7.28168×10^-14^
1.0	1.	1.27047×10^-7^	1.	3.54239×10^-9^	1.	4.32585×10^-15^

**Table 3:** HPM solutions along with absolute residuals for various γ when *M* = 1 and *R* = 0.3.

ξ	*γ* = 0.5		*γ* = 0.7		*γ* = 1.0	
	Solution	Residual	Solution	Solution	Residual	Solution
0.0	0.	0.	0.	0.	0.	0.
0.1	-0.00104157	4.04403×10^-8^	0.0551344	3.82927×10^-12^	0.0754476	1.49494×10^-12^
0.2	0.00408732	6.45840×10^-8^	0.11299	7.82407×10^-12^	0.152381	2.63267×10^-12^
0.3	0.0215448	6.60535×10^-8^	0.176287	1.09622×10^-11^	0.232285	2.95503×10^-12^
0.4	0.0574657	5.05202×10^-8^	0.247744	1.10414×10^-11^	0.316647	2.01172×10^-12^
0.5	0.117953	2.95181×10^-8^	0.330078	6.50968×10^-12^	0.406955	2.54463×10^-13^
0.6	0.209071	1.22993×10^-8^	0.426004	1.10312×10^-12^	0.5047	3.09242×10^-12^
0.7	0.336849	2.02592×10^-9^	0.538238	7.30838×10^-12^	0.611375	5.06706×10^-12^
0.8	0.507281	2.94278×10^-9^	0.669498	8.72680×10^-12^	0.728478	5.09570×10^-12^
0.9	0.726341	6.33995×10^-9^	0.822506	6.65201×10^-12^	0.857515	3.56648×10^-12^
1.0	1.	1.38222×10^-8^	1.	3.95861×10^-12^	1.	1.43396×10^-12^

**Table 4:** RK4 solutions along with absolute residuals for various *R* when *γ* = 1 and *R* = 0.3.

ξ	*R* = 0.7		*R* = 0.9		*R* = 1.0	
	Solution	Residual	Solution	Residual	Solution	Residual
0.0	0.	9.50912×10^-5^	0.	6.14718×10^-5^	0.	3.51857×10^-5^
0.1	0.0779056	5.47777×10^-6^	0.0758376	3.44574×10^-6^	0.074664	1.76807×10^-6^
0.2	0.157102	1.35127×10^-6^	0.153132	8.4865×10^-7^	0.150878	4.32163×10^-7^
0.3	0.23889	3.7631×10^-7^	0.23334	2.35587×10^-7^	0.230192	1.18306×10^-7^
0.4	0.32459	1.08892×10^-7^	0.317923	6.83416×10^-8^	0.314146	3.48009×10^-8^
0.5	0.415551	9.31167×10^-9^	0.408346	4.7683×10^-9^	0.404268	1.37043×10^-10^
0.6	0.513164	1.37754×10^-7^	0.50608	8.29938×10^-8^	0.502076	3.42575×10^-8^
0.7	0.618869	4.58691×10^-7^	0.612606	2.76956×10^-7^	0.609071	1.16329×10^-7^
0.8	0.734172	1.67895×10^-6^	0.729421	1.01238×10^-6^	0.726742	4.23736×10^-7^
0.9	0.860657	6.88124×10^-6^	0.858038	4.14347×10^-6^	0.856564	1.727×10^-6^
1.0	1.	1.43274×10^-4^	1.	8.5963×10^-5^	1.	3.5341×10^-5^

**Table 5:** RK4 solutions along with absolute residuals for various *M* when *γ* = 1 and *R* = 0.3.

ξ	*M* = 0.5		*M* = 0.7		*M* = 0.9	
	Solution	Residual	Solution	Residual	Solution	Residual
0.0	0.	3.64635×10^-6^	0.	3.64011×10^-6^	0.	4.86012×10^-6^
0.1	0.0734874	1.39958×10^-7^	0.0743047	1.68712×10^-7^	0.0750771	2.56125×10^-7^
0.2	0.148618	3.33737×10^-8^	0.150187	4.09526×10^-8^	0.151669	6.28066×10^-8^
0.3	0.227027	8.77935×10^-9^	0.229219	1.11017×10^-8^	0.231291	1.73203×10^-8^
0.4	0.310335	2.80348×10^-9^	0.312965	3.36487×10^-9^	0.315453	5.11758×10^-9^
0.5	0.400138	9.20462×10^-10^	0.402978	4.08603×10^-10^	0.405665	7.01896×10^-12^
0.6	0.498003	7.62562×10^-11^	0.500792	2.06073×10^-9^	0.503432	5.09238×10^-9^
0.7	0.605459	4.59149×10^-10^	0.607922	7.27692×10^-9^	0.610254	1.72149×10^-8^
0.8	0.723994	1.12162×10^-10^	0.72586	2.56535×10^-8^	0.727628	6.2306×10^-8^
0.9	0.855046	3.4671×10^-9^	0.856073	1.02751×10^-7^	0.857047	2.53755×10^-7^
1.0	1.	1.04217×10^-6^	1.	1.50015×10^-6^	1.	4.82583×10^-6^

**Table 6:** RK4 solutions along with absolute residuals for various *γ* when *Μ* = 1 and *R* = 0.3

ξ	*γ* = 0.5		*γ* = 0.7		*γ* = 1.0	
	Solution	Residual	Solution	Residual	Solution	Residual
0.0	0.	2.48394×10^-4^	0.	3.21426×10^-5^	0.	5.98185×10^-6^
0.1	-0.00104157	1.30619×10^-5^	0.0551344	1.7383×10^-6^	0.0754476	3.25803×10^-7^
0.2	0.00408732	3.20449×10^-6^	0.11299	4.27107×10^-7^	0.152381	8.00859×10^-8^
0.3	0.0215448	8.82603×10^-7^	0.176287	1.18155×10^-7^	0.232285	2.21753×10^-8^
0.4	0.0574657	2.56677×10^-7^	0.247744	3.46488×10^-8^	0.316647	6.51069×10^-9^
0.5	0.117953	1.1241×10^-8^	0.330078	1.03934×10^-9^	0.406955	1.84115×10^-10^
0.6	0.209071	2.90569×10^-7^	0.426004	3.78674×10^-8^	0.5047	7.08189×10^-9^
0.7	0.336849	9.76162×10^-7^	0.538238	1.27274×10^-7^	0.611375	2.37814×10^-8^
0.8	0.507281	3.57223×10^-6^	0.669498	4.63136×10^-7^	0.728478	8.64159×10^-8^
0.9	0.726341	1.46036×10^-5^	0.822506	1.8911×10^-6^	0.857515	3.52838×10^-7^
1.0	1.	3.09347×10^-4^	1.	3.76646×10^-5^	1.	6.92864×10^-6^

**Table 7:** Different order solutions along with absolute residuals when *R* = 1, *γ* = 1 and *Μ* = 3.

ξ	First Order		Third order		Fifth order	
	Solution	Residual	Solution	Solution	Residual	Solution
0.0	0.	0.	0.	0.	0.	0.
0.1	0.0746781	2.09932×10^-3^	0.0746634	3.0637×10^-6^	0.0746634	2.25321×10^-9^
0.2	0.0746781	3.77846×10^-3^	0.150878	4.79033×10^-6^	0.150878	2.65694×10^-9^
0.3	0.23023	4.70572×10^-3^	0.230192	4.54961×10^-6^	0.230192	9.23882×10^-10^
0.4	0.31419	4.71352×10^-3^	0.314146	2.7082×10^-6^	0.314146	1.56557×10^-9^
0.5	0.404314	3.84864×10^-3^	0.404268	3.42113×10^-7^	0.404268	3.09459×10^-9^
0.6	0.502119	2.38449×10^-3^	0.502076	1.42988×10^-6^	0.502076	2.99595×10^-9^
0.7	0.609108	7.83349×10^-4^	0.609071	2.05266×10^-6^	0.609071	1.86451×10^-9^
0.8	0.72677	4.0313×10^-4^	0.726742	1.6919×10^-6^	0.726742	7.09482×10^-10^
0.9	0.856579	7.06734×10^-4^	0.856564	8.84155×10^-7^	0.856564	1.02005×10^-10^
1.0	1.	0.	1.	0.	1.	8.59397×10^-21^

**Fig 2:**
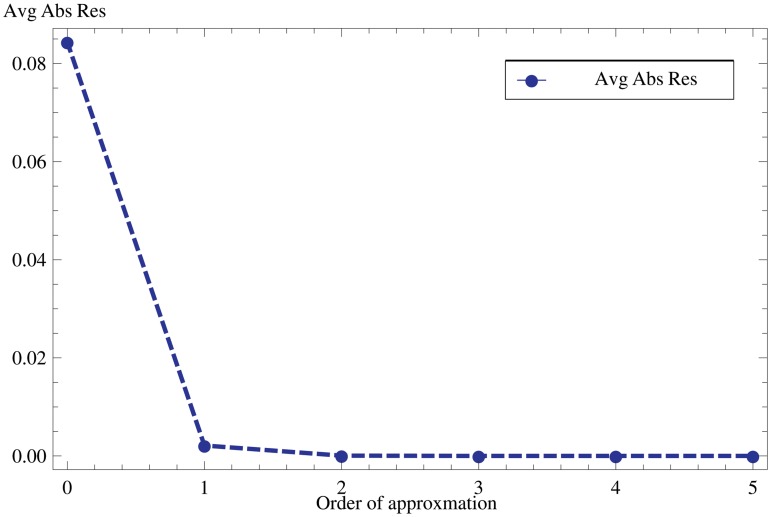
Convergence of HPM solution. Different order solutions along with absolute residuals shows the convergence of the HPM Solution.

We show the convergence of HPM solution in Fig. 2. This plot represents the average absolute residuals against different order approximations and it is clearly seen that HPM solution is convergent.

**Fig 3:**
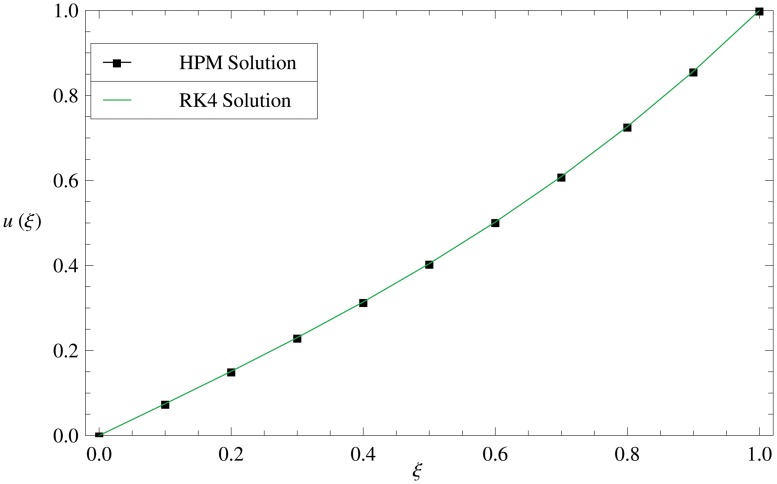
Comparison of HPM and RK4 solutions. Comparison of analytical and numerical solutions shows the validity of HPM solutions.

Validity of HPM solution is shown in Fig. 3, where we compare HPM and RK4 solutions for fixed values of *R*, *M* and *γ*, and observed that HPM solution is in high agreement with RK4 solution.

**Fig 4:**
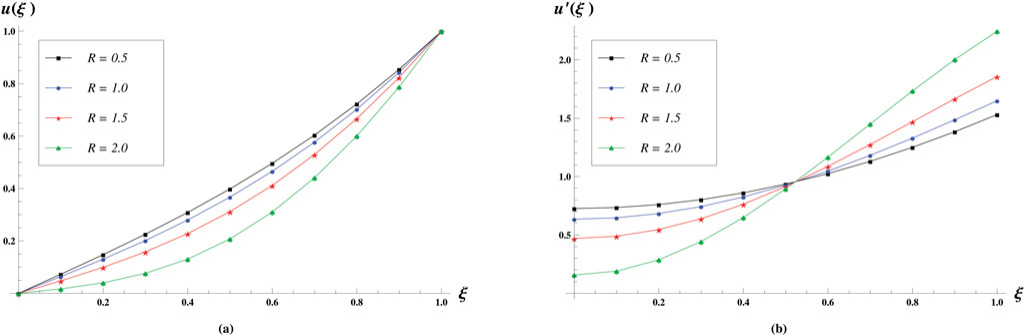
Velocity profiles for various values of *R* = 0.5,1,1.5,2 keeping *M* = 1 and *γ* = 1 fixed. The effect of Reynolds number *R* on the Normal velocity profiles is shown in (a) while the effect on the longitudinal velocity profiles is shown in (b).

The effect of the Reynolds number *R* on velocity profiles is shown in Fig. 4. In these profiles we varied *R* as *R* = 0.5,1,1.5,2 and observed that the normal velocity decreases with an increase in *R*. Also, the longitudinal velocity decreases near the central axis of the channel and increases near the plates. It has been analyzed that the normal velocity monotonically increases while longitudinal velocity monotonically decreases from ξ = 1 to ξ = 1 for fixed positive value of *R* at a given time.

**Fig 5:**
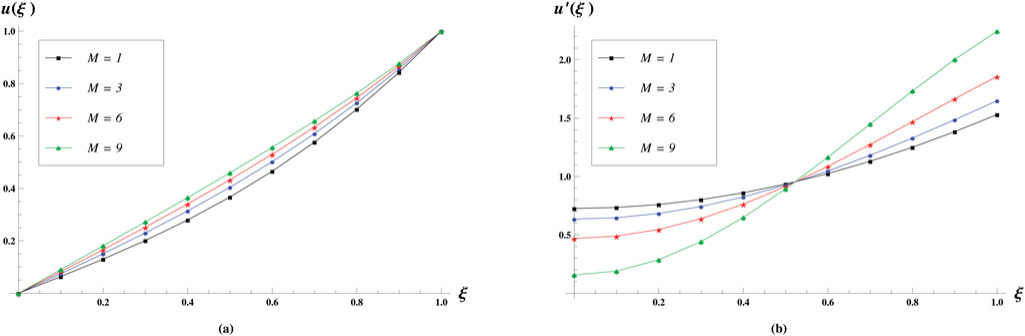
Velocity profiles for various values of *M* = 1,3,6,9 keeping *R* = 1 and *M* = 1 fixed. The effect of permeability constant *M* on the Normal velocity profiles is shown in (a) while the effect on the longitudinal velocity profiles is shown in (b).

Fig. 5 shows the effect of constant containing permeability *M* on the velocity profiles. In these profiles, we varied *M* as *M* = 1,3,6,9, and find that the normal velocity increases with the increase in *M* while longitudinal velocity increases near the central axis of the channel and decreases near the wall.

**Fig 6:**
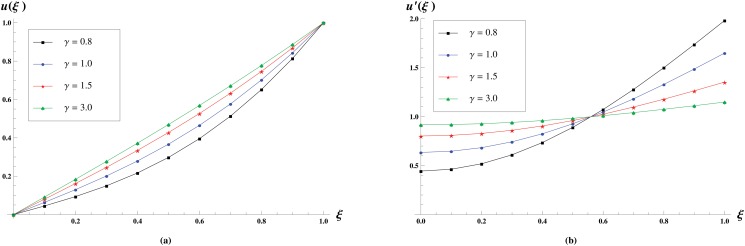
Velocity profiles for various values of *γ* = 0.8,1,1.5,3 keeping *R* = 1 and *γ* = 1 fixed. The effect of slip parameter *γ* on the Normal velocity profiles is shown in (a) while the effect on the longitudinal velocity profiles is shown in (b).

The effect of *γ* on the velocity profiles is depicted in Fig. 6. In these profiles we varied *γ* as *γ* = 0.8,1,1.5,3 and noted that normal velocity increases with the increase in *γ* whereas longitudinal velocity increases near the central axis of the channel and decreases near the plates.

**Fig 7:**
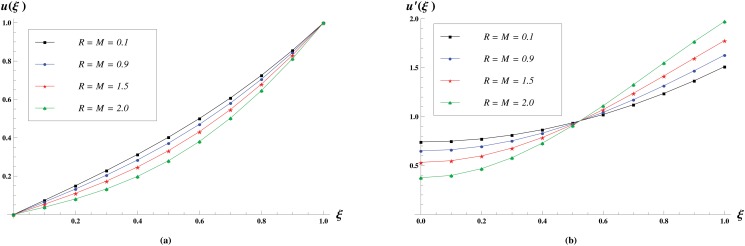
Velocity profiles for various values of *R = M* = 0.1,0.9,1.5,2 keeping *γ* = 1 fixed. The effect of Reynolds number *R* = *M* on the Normal velocity profiles is shown in (a) while the effect on the longitudinal velocity profiles is shown in (b).

The effect of *R* = *M* on the velocity profile is given in Fig. 7. In these profiles, we see that the normal velocity decreases with the increase in *R* = *M* while longitudinal velocity increases near the wall and decreases near the central axis of the channel.

S1, S2 and S3 Figs. depict the effects of *M* = *γ*, *R = γ and R* = *M* = *γ* on the velocity profiles respectively. In these profiles, we observed that normal velocity increases with the increase in *M* = *γ*, *R = γ and R* = *M* = *γ* respectively while longitudinal velocity decreases near the wall and increases near the central axis of the channel.

It can be observed from these profiles that similar behavior of normal and longitudinal velocity has been captured when we vary *M*,*γ*,*R = γ*,*M* = *γ and R* = *M* = *γ* while keeping other parameters fixed. It is also observed that *R* and *R = M* have a similar effect on the normal and longitudinal velocity profiles while keeping other parameters fixed.

## Conclusions

In this article, we find the similarity solution for an unsteady axisymmetric squeezing flow of incompressible Newtonian fluid through porous medium with slip boundary condition using HPM analytically and RK4 numerically. We determined the convergence of HPM solution using various order approximate solutions. In addition, we checked the validity of HPM solution by comparing analytical and numerical solutions. We observed some key findings related to the effects of dimensionless parameters on the velocity profiles. It was found that:

- The normal velocity decreases with the increase in Reynolds number *R*.
- With the increase in Reynolds number *R*, longitudinal velocity increases near the walls and decreases near the central axis of the channel.
- The normal velocity monotonically increases and the longitudinal velocity monotonically decreases from ξ = 0 to ξ = 1 for fixed positive value of *R* at any given time.
- *R* and *M* have opposite effects, while *γ* and *M* have similar effects on the normal and longitudinal velocity components.
- Similar velocity profiles are obtained when we vary *M*,*γ*,*R = γ*,*M* = *γ and R* = *M* = *γ*, while keeping the remaining parameters fixed.
- *R* and *R* = *M* have a similar effect on the velocity profiles, while keeping the remaining parameters fixed.

## Supporting Information

S1 Fig Velocity profiles for various values of *γ* = *M =* 0.8,1.2,1.5,2 keeping *R* = 1 fixed. The effect of *γ* = *M* on the Normal and longitudinal component of velocity profile is given in (a) and (b) respectively.
(EPS)

S2 Fig Velocity profiles for various values of *R* = *γ =* 0.6,0.9,1.2,2 keeping *M* = 3 fixed. The effect of *R* = *γ* on the Normal and longitudinal component of velocity profile is given in (a) and (b) respectively.
(EPS)

S3 Fig Velocity profiles for various values of *R* = *M* = *γ =* 0.7,1,1.2,2. The effect of *R* = *M* = *γ* on the Normal and longitudinal component of velocity profile is given in (a) and (b) respectively.
(EPS)

S1 Table Comparison of HPM and RK4 solutions for various *R* when *γ* = 1 and *M* = 3. (DOCX)

S2 Table Comparison of HPM and RK4 solutions for various *M* when *γ* = 1 and *R* = 03. (DOCX)

S3 Table Comparison of HPM and RK4 solutions for various *γ* when *M* = 1 and *R* = 0.3. (DOCX)
