# Correction: Modeling and Analysis of Unsteady Axisymmetric Squeezing Fluid Flow through Porous Medium Channel with Slip Boundary

**DOI:** 10.1371/journal.pone.0124851

**Published:** 2015-04-13

**Authors:** The PLOS ONE Staff

## Notice of Republication

This article was republished on March 20, 2015, to correct Figs 4, 5, 6, and 7, which were inadvertently cropped during the production process. In addition, a gamma character was missing from the legend of Table 3. The publisher apologizes for the errors. Please download this article again to view the correct version.

## Supporting Information

S1 File Originally published, uncorrected article. (PDF)

S2 File Republished, corrected article. (PDF)
