# Correction: Modeling and Analysis of Unsteady Axisymmetric Squeezing Fluid Flow through Porous Medium Channel with Slip Boundary

**DOI:** 10.1371/journal.pone.0125328

**Published:** 2015-04-23

**Authors:** The PLOS ONE Staff

There is an error in Fig 5(b). The authors have provided a corrected version here.

**Fig 5:**
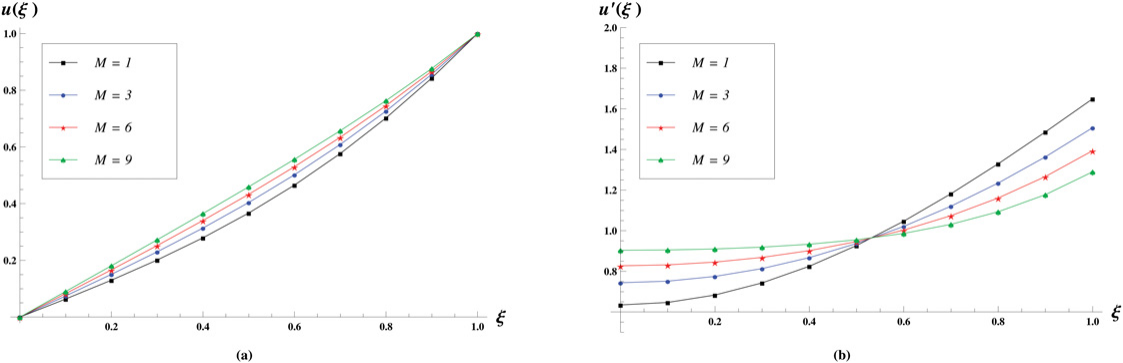
Velocity profiles for various values of *M* = 1,3,6,0 keeping R = 1 and γ = 1 fixed. The effect of permeability constant *M* on the Normal velocity profiles is shown in (a) while the effect on the longitudinal velocity profiles is shown in (b).

There are errors in the legends for Table 1, Table 3, Table 4, and S2 Table. The publisher apologizes for the errors.

**Table 1:** HPM solutions along with absolute residuals for various *R* when *γ* = 1 and *M* = 3.

*ξ*	*R* = 0.7		*R* = 0.9		*R* = 1.0	
	Solution	Residual	Solution	Residual	Solution	Residual
0.0	0.	0.	0.	0.	0.	0.
0.1	0.0779056	7.94082×10^-7^	0.0758376	1.07178×10^-9^	0.0746634	2025321×10^-9^
0.2	0.157102	1.2091×10^-6^	0.153132	1.88369×10^-9^	0.150878	2.65694×10^-9^
0.3	0.23889	9.05355×10^-7^	0.23334	2.10531×10^-9^	0.230192	9.23882×10^-10^
0.4	0.32459	3.44432×10^-7^	0.317923	1.41494×10^-9^	0.314146	1.56557×10^-9^
0.5	0.415552	2.53517×10^-6^	0.408346	2.23822×10^-10^	0.404268	3.09459×10^-9^
0.6	0.513164	5.28935×10^-6^	0.50608	2.26552×10^-9^	0.502076	2.99595×10^-9^
0.7	0.618869	7.77624×10^-6^	0.612606	3.67602×10^-9^	0.609071	1.86451×10^-9^
0.8	0.734172	8.81226×10^-6^	0.729421	3.68216×10^-9^	0.726742	7.09482×10^-10^
0.9	0.860657	7.25419×10^-6^	0.858038	2.57384×10^-9^	0.856564	1.02005×10^-10^
1.0	1.	2.67891×10^-6^	1.	1.04787×10^-9^	1.	8.59397×10^-21^

In the Table 1 legend, “*M* = 0.3” should read “*M* = 3.” Please see the correct Table 1 legend here.

A gamma is missing from the legend of Table 3. Please see the correct Table 3 legend here.

**Table 3:** HPM solutions along with absolute residuals for various *γ* when *M* = 1 and *R* = 0.3.

*ξ*	*γ* = 0.5		*γ* = 0.7		*γ* = 1.0	
	Solution	Residual	Solution	Residual	Solution	Residual
0.0	0.	0.	0.	0.	0.	0.
0.1	-0.00104157	4.04403×10^-8^	0.0551344	3.82927×10^-12^	0.0754476	1.49494×10^-12^
0.2	0.00408732	6.45840×10^-8^	0.11299	7.82407×10^-12^	0.152381	2.63267×10^-12^
0.3	0.0215448	6.60535×10^-8^	0.176287	1.09622×10^-11^	0.232285	2.95503×10^-12^
0.4	0.0574657	5.05202×10^-8^	0.247744	1.10414×10^-11^	0.316647	2.01172×10^-12^
0.5	0.117953	2.95181×10^-8^	0.330078	6.50968×10^-12^	0.406955	2.54463×10^-13^
0.6	0.209071	1.22993×10^-8^	0.426004	1.10312×10^-12^	0.5047	3.09242×10^-12^
0.7	0.336849	2.02592×10^-9^	0.538238	7.30838×10^-12^	0.611375	5.06706×10^-12^
0.8	0.507281	2.84278×10^-9^	0.669498	8.72680×10^-12^	0.728478	5.09570×10^-12^
0.9	0.726341	6.33995×10^-9^	0.822506	6.65201×10^-12^	0.857515	3.56648×10^-12^
1.0	1.	1.38222×10^-8^	1.	3.95861×10^-12^	1.	1.43396×10^-12^

In the legend of Table 4, “*R* = 0.3” should read “*M* = 3.” Please see the correct Table 4 legend here.

**Table 4:** RK4 solutions along with absolute residuals for various *R* when *γ* = 1 and *M = 3*.

*ξ*	*R* = 0.7		*R* = 0.9		*R* = 1.0	
	Solution	Residual	Solution	Residual	Solution	Residual
0.0	0.	9.50912×10^-5^	0.	6.14718×10^-5^	0.	3.53857×10^-5^
0.1	0.0779056	5.47777×10^-6^	0.0758376	3.44574×10^-6^	0.0746634	1.76807×10^-6^
0.2	0.157102	1.35127×10^-6^	0.153132	8.4865×10^-7^	0.150878	4032163×10^-7^
0.3	0.23889	3.7631×10^-7^	0.23334	2.35587×10^-7^	0.230192	1.18306×10^-7^
0.4	0.32459	1.08892×10^-7^	0.317923	6.83416×10^-8^	0.314146	3.48009×10^-8^
0.5	0.415551	9.31167×10^-9^	0.408346	4.7683×10^-9^	0.404268	1.37043×10^-10^
0.6	0.513164	1.37754×10^-7^	0.50608	8.29938×10^-8^	0.502076	3.42575×10^-8^
0.7	0.618869	4.58691×10^-7^	0.612606	2.76956×10^-7^	0.609071	1.16329×10^-7^
0.8	0.734172	1.67895×10^-6^	0.729421	10.1238×10^-6^	0.726742	4.23736×10^-7^
0.9	0.860657	6.88124×10^-6^	0.858038	4.14347×10^-6^	0.856564	1.727×10^-6^
1.0	1.	1.43274×10^-4^	1.	8.5963×10^-5^	1.	3.5341×10^-5^

In the legend of S2 Table, “*R* = 03” should read “*R* = 0.3.” Please see the correct S2 Table legend below.

## Supporting Information

S2 Table Comparison of HPM and RK4 solutions for various *M* when *γ* = 1 and *R* = 0.3. (DOCX)
