# Supplementary material for: Modeling and Analysis of Unsteady Axisymmetric Squeezing Fluid Flow through Porous Medium Channel with Slip Boundary
Source: PLoS One. 2015 Mar 4;10(3):e0117368. doi: 10.1371/journal.pone.0117368 (PMC4349699; doi:10.1371/journal.pone.0117368)
Supplement: S1 Table — (DOCX) [file pone.0117368.s004.docx]

**Table S1:** Comparison of HPM and RK4 solutions for various *R* when $\gamma=1\text{ and }M=3.$

| $\xi$ | $\left\vert HPM-RK4\right\vert$ $R=0.7$ $R=0.9$ $R=1.0$     | | |
| --- | --- | --- | --- |
| $0.0$ | $0.$ | $0.$ | $0.$ |
| $0.1$ | $1.3267\times10^{-8}$ | $2.31726\times10^{-12}$ | $4.5498\times10^{-12}$ |
| $0.2$ | $2.57913\times10^{-8}$ | $4.72244\times10^{-12}$ | $9.01579\times10^{-12}$ |
| $0.3$ | $3.67526\times10^{-8}$ | $7.20235\times10^{-12}$ | $1.31221\times10^{-11}$ |
| $0.4$ | $4.52127\times10^{-8}$ | $9.56535\times10^{-12}$ | $1.63655\times10^{-11}$ |
| $0.5$ | $5.01437\times10^{-8}$ | $1.14279\times10^{-11}$ | $1.817\times10^{-11}$ |
| $0.6$ | $5.05457\times10^{-8}$ | $1.22829\times10^{-11}$ | $1.81057\times10^{-11}$ |
| $0.7$ | $4.56514\times10^{-8}$ | $1.1656\times10^{-11}$ | $1.60312\times10^{-11}$ |
| $0.8$ | $3.5179\times10^{-8}$ | $9.28835\times10^{-12}$ | $1.20891\times10^{-11}$ |
| $0.9$ | $1.95554\times10^{-8}$ | $5.26348\times10^{-12}$ | $6.60919\times10^{-12}$ |
| $1.0$ | $3.8991\times10^{-15}$ | $8.32965\times10^{-15}$ | $2.22045\times10^{-16}$ |
