# Supplementary material for: Modeling and Analysis of Unsteady Axisymmetric Squeezing Fluid Flow through Porous Medium Channel with Slip Boundary
Source: PLoS One. 2015 Mar 4;10(3):e0117368. doi: 10.1371/journal.pone.0117368 (PMC4349699; doi:10.1371/journal.pone.0117368)
Supplement: S2 Table — (DOCX) [file pone.0117368.s005.docx]

**Table S2:** Comparison of HPM and RK4 solutions for various *M* when$\gamma=1\text{ and }R=0.3$.

| $\xi$ | $\left\vert HPM-RK4\right\vert$ $M=0.5$ $M=0.7$ $M=0.9$     | | |
| --- | --- | --- | --- |
| $0.0$ | $0.$ | $0.$ | $0.$ |
| $0.1$ | $1.8748\times10^{-9}$ | $6.01997\times10^{-11}$ | $2.9976\times10^{-15}$ |
| $0.2$ | $3.56557\times10^{-9}$ | $1.14026\times10^{-10}$ | $6.10623\times10^{-15}$ |
| $0.3$ | $4.90976\times10^{-9}$ | $1.56022\times10^{-10}$ | $8.99281\times10^{-15}$ |
| $0.4$ | $5.78338\times10^{-9}$ | $1.82327\times10^{-10}$ | $1.11022\times10^{-14}$ |
| $0.5$ | $6.11074\times10^{-9}$ | $1.90963\times10^{-10}$ | $1.24345\times10^{-14}$ |
| $0.6$ | $5.86584\times10^{-9}$ | $1.81717\times10^{-10}$ | $1.23235\times10^{-14}$ |
| $0.7$ | $5.06707\times10^{-9}$ | $1.55753\times10^{-10}$ | $1.09357\times10^{-14}$ |
| $0.8$ | $3.7679\times10^{-9}$ | $1.15114\times10^{-10}$ | $8.21565\times10^{-15}$ |
| $0.9$ | $2.04688\times10^{-9}$ | $6.22946\times10^{-11}$ | $4.4964\times10^{-15}$ |
| $1.0$ | $2.24478\times10^{-18}$ | $3.63331\times10^{-17}$ | $2.62114\times10^{-17}$ |
