# Supplementary material for: Modeling and Analysis of Unsteady Axisymmetric Squeezing Fluid Flow through Porous Medium Channel with Slip Boundary
Source: PLoS One. 2015 Mar 4;10(3):e0117368. doi: 10.1371/journal.pone.0117368 (PMC4349699; doi:10.1371/journal.pone.0117368)
Supplement: S3 Table — (DOCX) [file pone.0117368.s006.docx]

**Table S3:** Comparison of HPM and RK4 solutions for various $\gamma$ when$M=1\text{ and }R=0.3.$

| $\xi$ | $\left\vert HPM-RK4\right\vert$ $\gamma=0.5$ $\gamma=0.7$ $\gamma=1.0$     | | |
| --- | --- | --- | --- |
| $0.0$ | $0.$ | $0.$ | $0.$ |
| $0.1$ | $2.50704\times10^{-10}$ | $1.68754\times10^{-14}$ | $2.88658\times10^{-15}$ |
| $0.2$ | $4.76496\times10^{-10}$ | $3.15303\times10^{-14}$ | $5.88418\times10^{-15}$ |
| $0.3$ | $6.56322\times10^{-10}$ | $4.21885\times10^{-14}$ | $9.10383\times10^{-15}$ |
| $0.4$ | $7.75346\times10^{-10}$ | $4.70735\times10^{-14}$ | $1.23235\times10^{-14}$ |
| $0.5$ | $8.25205\times10^{-10}$ | $4.68514\times10^{-14}$ | $1.4877\times10^{-14}$ |
| $0.6$ | $8.0262\times10^{-10}$ | $4.16334\times10^{-14}$ | $1.59872\times10^{-14}$ |
| $0.7$ | $7.07421\times10^{-10}$ | $3.32512\times10^{-14}$ | $1.52656\times10^{-14}$ |
| $0.8$ | $5.40846\times10^{-10}$ | $2.33702\times10^{-14}$ | $1.21014\times10^{-14}$ |
| $0.9$ | $3.04464\times10^{-10}$ | $1.2268\times10^{-14}$ | $6.96665\times10^{-15}$ |
| $1.0$ | $4.14278\times10^{-13}$ | $2.64377\times10^{-16}$ | $1.63437\times10^{-17}$ |
