# Supplementary material for: Correction: Modeling and Analysis of Unsteady Axisymmetric Squeezing Fluid Flow through Porous Medium Channel with Slip Boundary
Source: PLoS One. 2015 Apr 13;10(4):e0124851. doi: 10.1371/journal.pone.0124851 (PMC4395451; doi:10.1371/journal.pone.0124851)
Supplement: S2 File — (PDF) [file pone.0124851.s002.pdf]

PLOS | ONE

RESEARCH ARTICLE

Modeling and Analysis of Unsteady Axisymmetric Squeezing Fluid Flow through Porous Medium Channel with Slip Boundary

Mubashir Qayyum*, **Hamid Khan, M. Tariq Rahim, Inayat Ullah**

Department of Mathematics, National University of Computer & Emerging Sciences - FAST Peshawar Campus, Peshawar, 25000, Pakistan

* mubashir.qayyum@nu.edu.pk

Abstract

The aim of this article is to model and analyze an unsteady axisymmetric flow of non-conducting, Newtonian fluid squeezed between two circular plates passing through porous medium channel with slip boundary condition. A single fourth order nonlinear ordinary differential equation is obtained using similarity transformation. The resulting boundary value problem is solved using Homotopy Perturbation Method (HPM) and fourth order Explicit Runge Kutta Method (RK4). Convergence of HPM solution is verified by obtaining various order approximate solutions along with absolute residuals. Validity of HPM solution is confirmed by comparing analytical and numerical solutions. Furthermore, the effects of various dimensionless parameters on the longitudinal and normal velocity profiles are studied graphically.

OPEN ACCESS

Citation: Qayyum M, Khan H, Rahim MT, Ullah I (2015) Modeling and Analysis of Unsteady Axisymmetric Squeezing Fluid Flow through Porous Medium Channel with Slip Boundary. PLoS ONE 10(3): e0117368. doi:10.1371/journal.pone.0117368

Academic Editor: Ming Dao, Massachusetts Institute Of Technology, UNITED STATES

Received: July 12, 2014

Accepted: December 22, 2014

Published: March 4, 2015

Copyright: © 2015 Qayyum et al. This is an open access article distributed under the terms of the Creative Commons Attribution License, which permits unrestricted use, distribution, and reproduction in any medium, provided the original author and source are credited.

Data Availability Statement: All relevant data are within the paper and its Supporting Information files.

Funding: The authors received no specific funding for this work.

Competing Interests: The authors have declared that no competing interests exist.

Introduction

The interest in behavior of fluid flow through porous media began in the early days of oil and gas production, where the focus was on estimating and optimizing production. Similarly, another important application is the simulation of ground water pollution, mostly occurring due to leakage of chemicals from tanks and oil pipelines. The objective is to consider groundwater as one medium and polluted water as another, so that the spreading in the latter medium and its consequences can be studied.

In recent times, after the introduction of the modified Darcy Law [1], analysis through porous medium has been an important topic for the research community, as it finds is use in fields such as reservoir, petroleum, chemical, civil, environmental, agricultural, and biomedical engineering. Some practical applications in these fields include chemical reactors, filtration, geothermal reservoirs, ground water hydrology, drainage and recovery of crude oil from pores of reservoir rocks [2–7].

Squeezing flow has attracted significant attention because of its broad applications in many fields such as chemical, mechanical, and industrial engineering, and in bio-mechanics and food industries. Practical applications of squeezing flows in these fields are polymer processing,

modeling of lubrication systems, and compression and injection molding, etc. These flows are induced by applying normal stresses or vertical velocities by means of a moving boundary, which can be frequently observed in various hydro-dynamical tools and machines.

Pioneering work on squeezing flows was investigated by Stefan [8] in which he proposed an adhoc asymptotic solution of Newtonian fluid. A solution considering inertial terms was found by Thorp [9]. However, Gupta and Gupta [10] later showed that this solution failed to satisfy boundary conditions. The effect of the inertial term in squeezing films between circular plates has been evaluated by Kuzma [11]. Elkouh [12] studied the squeeze film between two plane annuli taking fluid inertia effects under consideration. Verma [13] and Singh et al. [14] set up numerical solutions of the squeezing flows between parallel plates. Leider and Bird [15] carried out theoretical analysis for squeezing flow of power-law fluid between parallel plates. Naduvinamani et al. [16] investigated squeeze film lubrication of a short porous journal with couple stress fluids. Steady axisymmetric squeezing fluid flow in a porous medium has been analyzed by Islam et al. [17]. Hamza [18] worked on squeeze films considering MHD effect. Suction and injection effects on the flow of electrically conducting viscous fluid squeezed between two parallel disks was studied by Domairry et al. [19]. The study of the porosity and squeezing effects, while investigating the unsteady squeezing flow of visco-elastic Jeffery fluid between parallel disks, has been performed by Qayyum et al. [20]. Apart from the mentioned scholars, other researchers have also carried out different theoretical and experimental studies of squeezing flows [21–24].

No-slip boundary condition is one of the main concepts of fluid dynamics. Consider a liquid flowing over a solid wall. The condition in which the liquid molecules near the solid wall are motionless, relative to the wall, is called no-slip boundary [25]. This boundary condition has been employed in modeling various viscous and visco-elastic fluid flow problems. Firstly, Navier [26] proposed the general boundary condition which shows fluid slip at the liquid-solid interface. According to him, the difference between the boundary and fluid velocities is proportional to the shear stress at the boundary. The dimension of proportionality constant is length, and this is known as the slip parameter. There are numerous situations in which no-slip boundary condition is not appropriate. For instance, flow on multiple interfaces, polymeric liquids when the weight of the molecules is high, fluids containing concerted suspensions, and thin film problems.

A number of perturbation techniques which can solve non-linear boundary value problems analytically are discussed in literature. But the assumption of small parameter is a limitation in these techniques. Recently, a technique was proposed by He [27–30], that combines homotopy and the traditional perturbation method [31– 34]. This technique was the beginning of homotopy perturbation method (HPM). In a series of papers, He applied this method to discuss nonlinear boundary value problems [27–30]. As a result, many researchers have used HPM to solve non-linear differential equations in different fields as it is not only easy to use, but also successful. This method minimizes the limitations commonly associated with perturbation techniques, while taking full advantage of the traditional perturbation methods. In fluid dynamics, Siddiqui et al. [35, 36] applied this technique for solving non-linear boundary value problems arising in Newtonian and non-Newtonian fluids. In addition, Zhou and Wu [37] used this technique in an inverse heat problem. Also, Hamid et al. [38] compared the method with other analytical and numerical techniques, while solving higher order non-linear differential equations.

The objective of this manuscript is to use HPM for the solution of an unsteady axisymmetric squeezing fluid flow between two circular plates through porous medium with slip boundary condition. Validity of HPM solution is confirmed by comparing analytical and numerical solutions. In addition, effects of different dimensionless parameters on the velocity profiles are studied graphically.

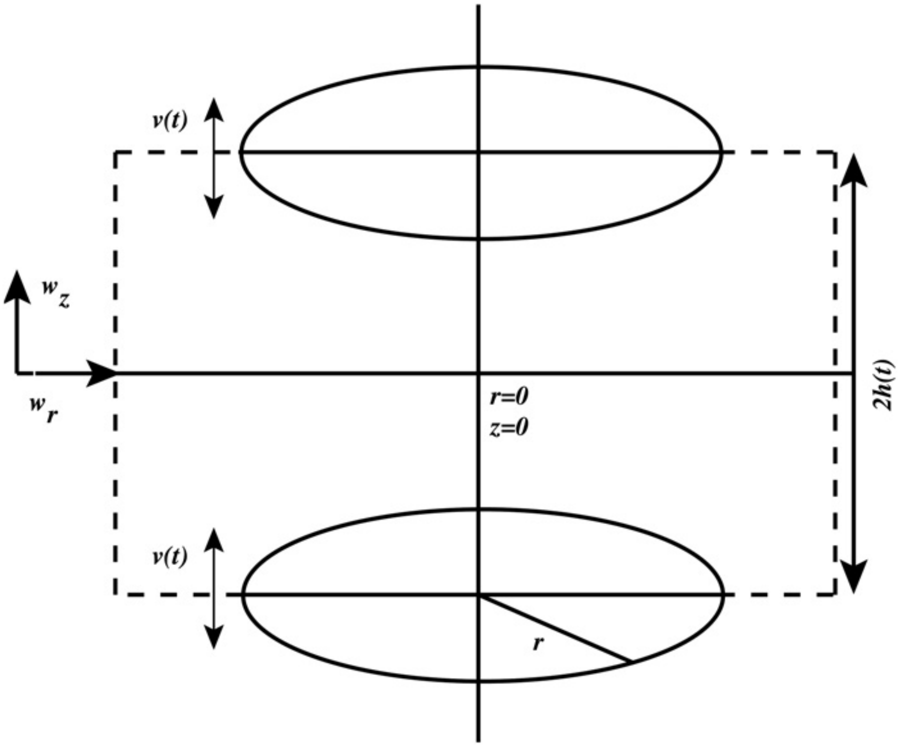

Fig 1. Geometry of the flow. Squeezing flow between two circular plates having distance $2h(t)$. r-axis is the central axis of the channel while z-axis is taken normal to it. The plates move symmetrically with respect to $z = 0$ at a speed $v(t)$ while the flow is axisymmetric about $r = 0$. Longitudinal and normal velocity components in radial and axial directions are $w_r(r,z,t)$ and $w_z(r,z,t)$ respectively.

doi:10.1371/journal.pone.0117368.g001

Description of the Problem

An unsteady axisymmetric squeezing flow of incompressible first grade fluid with density ρ, viscosity μ, and kinematic viscosityυ, squeezed between two circular plates having speed $v(t)$ and passing through porous medium channel is considered. It is assumed that at any time t, the distance between the two circular plates is $2h(t)$. Also, it is assumed that r-axis is the central axis of the channel while z-axis is taken normal to it. Plates move symmetrically with respect to the central axis $z = 0$ while the flow is axisymmetric about $r = 0$. The longitudinal and normal velocity components in radial and axial directions are $w_r(r,z,t)$ and $w_z(r,z,t)$ respectively. The geometrical representation of the flow is illustrated in Fig. 1.

Problem Formulation

The basic governing equations of motion are

$$\nabla \cdot W = 0 \tag{1}$$

$$\rho\left[\frac{\partial W}{\partial t} + (W \cdot \nabla)W\right] = \rho \mathrm{f} + \nabla \cdot T + \tilde{r} \tag{2}$$

where

$$T = -pI + \mu A \tag{3}$$

$$A = \nabla W + (\nabla W)^t \tag{4}$$

and **W** is the velocity vector, p is the pressure, f is the body force, **T** is the Cauchy stress tensor, **A** is the Rivlin-Ericksen tensor, μ is the coefficient of viscosity, and $\tilde{r}$ is the Darcy's resistance. According to Breugem equation [39], $\tilde{r}$can be written as:

$$\tilde{r} = -\frac{\mu}{k} W \tag{5}$$

where k is the permeability constant.

Now, we formulate the unsteady two-dimensional flow through porous medium. After neglecting body force we assume that

$$W = [w_r(r, z, t), 0, w_z(r, z, t)] \tag{6}$$

and introduce the vorticity function Ω (r,z,t)and generalized pressure $\widehat{P}(r, z, t)$as

$$\Omega(r, z, t) = \frac{\partial w_z}{\partial r} - \frac{\partial w_r}{\partial z} \tag{7}$$

$$\widehat{P}(r, z, t) = \frac{\rho}{2}\left[w_r^2 + w_z^2\right] + p \tag{8}$$

Equations (1) and (2) can then be reduced to

$$\frac{\partial w_r}{\partial r} + \frac{w_r}{r} + \frac{\partial w_z}{\partial z} = 0 \tag{9}$$

$$\frac{\partial \widehat{P}}{\partial r} + \rho\left(\frac{\partial w_r}{\partial t} - w_z\Omega\right) = -\mu\left(\frac{\partial \Omega}{\partial z} + \frac{w_r}{k}\right) \tag{10}$$

$$\frac{\partial \widehat{P}}{\partial z} + \rho\left(\frac{\partial w_z}{\partial t} + w_r\Omega\right) = \mu\left(\frac{1}{r}\frac{\partial}{\partial r}(r\Omega) - \frac{w_z}{k}\right) \tag{11}$$

The boundary conditions on $w_r(r,z,t)$ and $w_z(r,z,t)$ are

$$\begin{aligned} w_r(r, z, t) = \beta\frac{\partial}{\partial z}w_r(r, z, t)\text{and}w_z(r, z, t) = v(t)\text{at}z = h \\ \frac{\partial}{\partial z}w_r(r, z, t) = 0\text{and}w_z(r, z, t) = 0\text{at}z = 0 \end{aligned} \tag{12}$$

where $v(t) = \frac{dh}{dt}$ is the velocity of the plates. The boundary conditions in (12) are due to slip at the upper plate when z = h and symmetry at z = 0. If we launch the dimensionless parameter

$$\xi = \frac{z}{h(t)} \tag{13}$$

Equations (7),(9),(10)and(11)are converted to

$$\Omega(r,z,t) = \frac{\partial w_z}{\partial r} - \frac{1}{h}\frac{\partial w_r}{\partial \xi} \tag{14}$$

$$\frac{\partial w_r}{\partial r} + \frac{w_r}{r} + \frac{1}{h}\frac{\partial w_z}{\partial \xi} = 0 \tag{15}$$

$$\frac{\partial \widehat{P}}{\partial r} + \rho\left(\frac{\partial w_r}{\partial t} - w_z\Omega\right) = -\mu\left(\frac{1}{h}\frac{\partial \Omega}{\partial \xi} + \frac{w_r}{k}\right) \tag{16}$$

$$\frac{1}{h}\frac{\partial \widehat{P}}{\partial \xi} + \rho\left(\frac{\partial w_z}{\partial t} + w_r\Omega\right) = \mu\left(\frac{1}{r}\frac{\partial}{\partial r}(r\Omega) - \frac{w_z}{k}\right) \tag{17}$$

The boundary conditions on w_r and w_z are

$$\begin{aligned} w_r &= \beta\frac{1}{h}\frac{\partial w_r}{\partial \xi}\text{and}w_z = v(t)\text{at}\xi = 1 \\ \frac{\partial w_r}{\partial \xi} &= 0\text{and}w_z = 0\text{at}\xi = 0. \end{aligned} \tag{18}$$

After eliminating the $\widehat{P}(r,z,t)$ between (16) and (17), we obtain:

$$\rho\left[\frac{\partial \Omega}{\partial t} + w_r\frac{\partial \Omega}{\partial r} + \frac{w_z}{h}\frac{\partial \Omega}{\partial \xi} - \frac{w_r}{r}\Omega\right] = \mu\left[\nabla^2\Omega - \left(\frac{1}{r^2} + \frac{1}{k}\right)\Omega\right] \tag{19}$$

where $\bigtriangledown^2$ is the Laplacian operator.

Defining velocity components as [11]

$$\begin{aligned} w_r &= -\frac{r}{2h(t)}v(t)F'(\xi) \\ w_z &= v(t)F(\xi) \end{aligned} \tag{20}$$

we see that (15) is identically satisfied and therefore, (19) becomes

$$\frac{d^4F}{d\xi^4} + R\left[(\xi - F)\frac{d^3F}{d\xi^3} + 2\frac{d^2F}{d\xi^2}\right] - Q\frac{d^2F}{d\xi^2} - M\frac{d^2F}{d\xi^2} = 0 \tag{21}$$

where

$$R = \frac{h\,v(t)}{\upsilon}, Q = \frac{h^2}{\upsilon v(t)}\frac{dv(t)}{dt}\,\text{and}\,M = \frac{h^2}{k} \tag{22}$$

Both R and Q are functions of time but for similarity solution we consider R and Q constants. Since$v = \frac{dh}{dt}$, integrating the first equation of (22), we obtain:

$$h(t) = (Ct + D)^{\frac{1}{2}} \tag{23}$$

where C and D are constants. When $C>0$ and $D>0$, the plates move away from each other symmetrically with respect to ξ. The squeezing flow exists when the plates approach each other when $C>0$, $D>0$ and $h(t)>0$. From (22) and (23) it follows that $Q = -R$. Then (21) becomes

$$\frac{d^4F}{d\xi^4} + R\left[(\xi - F)\frac{d^3F}{d\xi^3} + 3\frac{d^2F}{d\xi^2}\right] - M\frac{d^2F}{d\xi^2} = 0 \tag{24}$$

After using (18) and (20), we establish the following boundary conditions in case of slip at the upper plate:

$$\begin{aligned} F(1) = 1, F'(1) = \gamma F''(1) \\ F(0) = 0, F''(0) = 0 \end{aligned} \tag{25}$$

Fundamental Theory of HPM [27–30]

To exhibit the basic theory of HPM, let us consider the following differential equation:

$$\begin{aligned} L(w) + N(w) - g(r) = 0, \quad r \in \Omega \\ B\left(w, \frac{dw}{dn}\right) = 0, \quad r \in \Upsilon \end{aligned} \tag{26}$$

where w is an unknown function and $g(r)$ is a known function. L, N, B are linear, nonlinear and boundary operators respectively. Also Υ is the boundry of the domain Ω.

We construct Homotopy $\theta(r, p) : \Omega \times [0, 1] \rightarrow \mathbb{R}$ which satisfies

$$\psi(\theta, p) = (1 - p)[L(\theta) - L(w_0)] + p[L(\theta) + N(\theta) - g(r)] = 0, r \in \Omega \tag{27}$$

where p ε [0, 1] is an embedding parameter, and w_0 is the initial guess of (26) which satisfies the boundary conditions. From (27), we have:

$$\begin{aligned} \psi(\theta, 0) = L(\theta) - L(w_0) = 0 \\ \psi(\theta, 1) = L(\theta) + N(\theta) - g(r) = 0 \end{aligned} \tag{28}$$

Thus, as p varies from 0 to1, the solution $\theta(r,p)$ approaches from w_0(r) to$\tilde{w}(r)$.

To obtain an approximate solution, we expand $\theta(r,p)$ in a Taylor series about p as follows: $\theta(r, p) = \theta_0 + \sum_{k=1}^{\infty} \theta_k p^k$ Setting p = 1, the approximate solution of (26) would be

$$\tilde{w} = \lim_{p \rightarrow 1} \theta(r, p) = \sum_{k=1}^{\infty} \theta_k \tag{29}$$

Application of HPM

Using (24) and (25), various order problems are as follows:Zeroth-Order Problem

$$\begin{aligned} u_0^{(iv)}(\xi) = 0, \\ u_0(0) = 0, u''_0(0) = 0, u_0(1) = 1, u'_0(1) = \gamma u''_0(1) \end{aligned} \tag{30}$$

First-Order Problem

$$\begin{aligned} u_1^{(iv)}(\xi) - Mu''_0(\xi) + 3Ru''_0(\xi) + R\xi u'''_0(\xi) - Ru_0(\xi)u'''_0(\xi) = 0, \\ u_1(0) = 0, u''_1(0) = 0, u_1(1) = 0, u'_1(1) = \gamma u''_1(1) \end{aligned} \tag{31}$$

Second-Order Problem

$$\begin{aligned} u_2^{(iv)}(\xi) - Mu''_1(\xi) + 3Ru''_1(\xi) - Ru_1(\xi)u'''_0(\xi) + R\xi u'''_1(\xi) - Ru_0(\xi)u'''_1(\xi) = 0, \\ u_2(0) = 0, u''_2(0) = 0, u_2(1) = 0, u'_2(1) = \gamma u''_2(1) \end{aligned} \tag{32}$$

Third-Order Problem

$$\begin{gathered} u_3^{(iv)}(\xi) - Mu''_2(\xi) + 3Ru''_2(\xi) - Ru_2(\xi)u'''_0(\xi) - Ru_1(\xi)u'''_1(\xi) + R\xi u'''_2(\xi) \\ -Ru_0(\xi)u'''_2(\xi) = 0, \\ u_3(0) = 0, u''_3(0) = 0, u_3(1) = 0, u'_3(1) = \gamma u''_3(1) \end{gathered} \tag{33}$$

Fourth-Order Problem

$$\begin{gathered} u_4^{(iv)}(\xi) - Mu''_3(\xi) + 3Ru''_3(\xi) - Ru_3(\xi)u'''_0(\xi) - Ru_2(\xi)u'''_1(\xi) - Ru_1(\xi)u'''_2(\xi) \\ +R\xi u'''_3(\xi) - Ru_0(\xi)u'''_3(\xi) = 0, \\ u_4(0) = 0, u''_4(0) = 0, u_4(1) = 0, u'_4(1) = \gamma u''_4(1) \end{gathered} \tag{34}$$

Fifth-Order Problem

$$\begin{gathered} u_5^{(iv)}(\xi) - Mu''_4(\xi) + 3Ru''_4(\xi) - Ru_4(\xi)u'''_0(\xi) - Ru_3(\xi)u'''_1(\xi) - Ru_2(\xi)u'''_2(\xi) \\ -Ru_1(\xi)u'''_3(\xi) + R\xi u'''_4(\xi) - Ru_0(\xi)u'''_4(\xi) = 0, \\ u_5(0) = 0, u''_5(0) = 0, u_5(1) = 0, u'_5(1) = \gamma u''_5(1) \end{gathered} \tag{35}$$

By considering fifth order solution, we have

$$\tilde{u}(\xi) = \sum_{i=0}^{5} u_i(\xi) \tag{36}$$

Keeping $R = 1$, $M = 1$ and γ, the approximate solution is

$$\tilde{u}(\xi) = \left\{ \begin{gathered} 0.634674\xi + 0.409456\xi^3 - 0.0482189\xi^5 + 0.00465172\xi^7 - 0.000635324\xi^9 \\ +0.0000811794\xi^{11} - 9.86308 \times 10^{-6}\xi^{13} + 1.00747 \times 10^{-6}\xi^{15} - 8.04603 \times 10^{-8}\xi^{17} \\ +4.41816 \times 10^{-9}\xi^{19} - 1.36769 \times 10^{-10}\xi^{21} + 1.54292 \times 10^{-12}\xi^{23} \end{gathered} \right\} \tag{37}$$

The residual of the problem is

$$\Re = \frac{d^4\tilde{u}}{d\xi^4} + R\left[(\xi - \tilde{u})\frac{d^3\tilde{u}}{d\xi^3} + 3\frac{d^2\tilde{u}}{d\xi^2}\right] - M\frac{d^2\tilde{u}}{d\xi^2} \tag{38}$$

If $\Re = 0$, then $\tilde{u}$ will be the exact solution, but usually this does not occur in non-linear problems.

Results and Discussions

In the present article, we considered an unsteady axisymmetric squeezing flow of incompressible Newtonian fluid passing through porous medium with slip boundary condition. The resulting non-linear boundary value problem is solved through HPM and RK4.

There are three parameters; Reynolds number R, constant containing permeability M, and slip parameter γ in the current problem. We present our discussion of results based on different compositions of these parameters. First of all we solve the problem for various values of R, M and γ analytically using HPM. This is illustrated in Tables 1, 2, and 3. Secondly, we solve the problem numerically using RK4 for various R,M and γ. This is explained in Tables 4, 5, and 6. We also check the convergence of HPM solution using different order approximations in Table 7. Finally, we check the validity of HPM solutions by comparing analytical and numerical solutions. This is demonstrated in S1, S2 and S3 Table. All the tables signify the efficiency of HPM. Furthermore, we investigated the effects of various dimensionless parameters on the normal and longitudinal velocity profiles graphically.

Table 1. HPM solutions along with absolute residuals for various *R* when γ = 1 and *M* = 0.3.

ξ	*R* = 0.7		*R* = 0.9		*R* = 1.0	
	Solution	Residual	Solution	Residual	Solution	Residual
0.0	0.	0.	0.	0.	0.	0.
0.1	0.0779056	7.94082×10^{-7}	0.0758376	1.07178×10^{-9}	0.0746634	2.25321×10^{-9}
0.2	0.157102	1.2091×10^{-6}	0.15132	1.88369×10^{-9}	0.150878	2.65694×10^{-9}
0.3	0.23889	9.05355×10^{-7}	0.23334	2.10531×10^{-9}	.0230192	9.23882×10^{-10}
0.4	0.32459	3.44432×10^{-7}	0.317923	1.41494×10^{-9}	0.314146	1.56557×10^{-9}
0.5	0.415552	2.53517×10^{-6}	0.408346	2.23822×10^{-10}	0.404268	3.09459×10^{-9}
0.6	0.513164	5.28935×10^{-6}	0.50608	2.26552×10^{-9}	0.502076	2.99595×10^{-9}
0.7	0.618869	7.77624×10^{-6}	0.612606	3.67602×10^{-9}	0.609071	1.86451×10^{-9}
0.8	0.734172	8.81226×10^{-6}	0.729421	3.68216×10^{-9}	0.726742	7.09482×10^{-10}
0.9	0.860657	7.25419×10^{-6}	0.858038	2.57384×10^{-9}	0.856564	1.02005×10^{-10}
1.0	1.	2.67891×10^{-6}	1.	1.04787×10^{-9}	1.	8.59397×10^{-21}

doi:10.1371/journal.pone.0117368.t001

Table 2. HPM solutions along with absolute residuals for various *M* when γ = 1 and *R* = 0.3.

ξ	*M* = 0.5		*M* = 0.7		*M* = 0.9	
	Solution	Residual	Solution	Solution	Residual	Solution
0.0	0.	0.	0.	0.	0.	0.
0.1	0.0734874	2.1267×10^{-7}	0.0743047	9.40321×10^{-9}	0.0750771	1.62039×10^{-12}
0.2	0.148618	3.80716×10^{-7}	0.150187	1.62781×10^{-8}	0.151669	1.91338×10^{-12}
0.3	0.227027	4.7353×10^{-7}	0.229219	1.90699×10^{-8}	0.231291	6.71317×10^{-13}
0.4	0.310335	4.83136×10^{-7}	0.312965	1.77119×10^{-8}	0.315453	1.11729×10^{-12}
0.5	0.400138	4.24408×10^{-7}	0.402978	1.34725×10^{-8}	0.405665	2.21927×10^{-12}
0.6	0.498003	3.27592×10^{-7}	0.500792	8.27910×10^{-9}	0.503432	2.15310×10^{-12}
0.7	0.605459	2.26980×10^{-7}	0.607922	3.91263×10^{-9}	0.610254	1.34291×10^{-12}
0.8	0.723994	1.50704×10^{-7}	0.72586	1.47900×10^{-9}	0.727628	5.12541×10^{-13}
0.9	0.855046	1.15451×10^{-7}	0.856073	1.35555×10^{-9}	0857047	7.28168×10^{-14}
1.0	1.	1.27047×10^{-7}	1.	3.54239×10^{-9}	1.	4.32585×10^{-15}

doi:10.1371/journal.pone.0117368.t002

Table 3. HPM solutions along with absolute residuals for various γ when *M* = 1 and *R* = 0.3.

ξ	γ = 0.5		γ = 0.7		γ = 1.0	
	Solution	Residual	Solution	Solution	Residual	Solution
0.0	0.	0.	0.	0.	0.	0.
0.1	-0.00104157	4.04403×10^{-8}	0.0551344	3.82927×10^{-12}	0.0754476	1.49494×10^{-12}
0.2	0.00408732	6.45840×10^{-8}	0.11299	7.82407×10^{-12}	0.152381	2.63267×10^{-12}
0.3	0.0215448	6.60535×10^{-8}	0.176287	1.09622×10^{-11}	0.232285	2.95503×10^{-12}
0.4	0.0574657	5.05202×10^{-8}	0.247744	1.10414×10^{-11}	0.316647	2.01172×10^{-12}
0.5	0.117953	2.95181×10^{-8}	0.330078	6.50968×10^{-12}	0.406955	2.54463×10^{-13}
0.6	0.209071	1.22993×10^{-8}	0.426004	1.10312×10^{-12}	0.5047	3.09242×10^{-12}
0.7	0.336849	2.02592×10^{-9}	0.538238	7.30838×10^{-12}	0.611375	5.06706×10^{-12}
0.8	0.507281	2.94278×10^{-9}	0.669498	8.72680×10^{-12}	0.728478	5.09570×10^{-12}
0.9	0.726341	6.33995×10^{-9}	0.822506	6.65201×10^{-12}	0.857515	3.56648×10^{-12}
1.0	1.	1.38222×10^{-8}	1.	3.95861×10^{-12}	1.	1.43396×10^{-12}

doi:10.1371/journal.pone.0117368.t003

Table 4. RK4 solutions along with absolute residuals for various *R* when *γ* = 1 and *R* = 0.3.

ξ	*R* = 0.7		*R* = 0.9		*R* = 1.0	
	Solution	Residual	Solution	Residual	Solution	Residual
0.0	0.	9.50912×10^{-5}	0.	6.14718×10^{-5}	0.	3.51857×10^{-5}
0.1	0.0779056	5.47777×10^{-6}	0.0758376	3.44574×10^{-6}	0.074664	1.76807×10^{-6}
0.2	0.157102	1.35127×10^{-6}	0.153132	8.4865×10^{-7}	0.150878	4.32163×10^{-7}
0.3	0.23889	3.7631×10^{-7}	0.23334	2.35587×10^{-7}	0.230192	1.18306×10^{-7}
0.4	0.32459	1.08892×10^{-7}	0.317923	6.83416×10^{-8}	0.314146	3.48009×10^{-8}
0.5	0.415551	9.31167×10^{-9}	0.408346	4.7683×10^{-9}	0.404268	1.37043×10^{-10}
0.6	0.513164	1.37754×10^{-7}	0.50608	8.29938×10^{-8}	0.502076	3.42575×10^{-8}
0.7	0.618869	4.58691×10^{-7}	0.612606	2.76956×10^{-7}	0.609071	1.16329×10^{-7}
0.8	0.734172	1.67895×10^{-6}	0.729421	1.01238×10^{-6}	0.726742	4.23736×10^{-7}
0.9	0.860657	6.88124×10^{-6}	0.858038	4.14347×10^{-6}	0.856564	1.727×10^{-6}
1.0	1.	1.43274×10^{-4}	1.	8.5963×10^{-5}	1.	3.5341×10^{-5}

doi:10.1371/journal.pone.0117368.t004

We show the convergence of HPM solution in Fig. 2. This plot represents the average absolute residuals against different order approximations and it is clearly seen that HPM solution is convergent.

Validity of HPM solution is shown in Fig. 3, where we compare HPM and RK4 solutions for fixed values of R, M and γ, and observed that HPM solution is in high agreement with RK4 solution.

The effect of the Reynolds number R on velocity profiles is shown in Fig. 4. In these profiles we varied R as $R = 0.5,1,1.5,2$ and observed that the normal velocity decreases with an increase in R. Also, the longitudinal velocity decreases near the central axis of the channel and increases near the plates. It has been analyzed that the normal velocity monotonically increases while longitudinal velocity monotonically decreases from $\xi = 1$ to $\xi = 1$ for fixed positive value of R at a given time.

Table 5. RK4 solutions along with absolute residuals for various *M* when *γ* = 1 and *R* = 0.3.

ξ	*M* = 0.5		*M* = 0.7		*M* = 0.9	
	Solution	Residual	Solution	Residual	Solution	Residual
0.0	0.	3.64635×10^{-6}	0.	3.64011×10^{-6}	0.	4.86012×10^{-6}
0.1	0.0734874	1.39958×10^{-7}	0.0743047	1.68712×10^{-7}	0.0750771	2.56125×10^{-7}
0.2	0.148618	3.33737×10^{-8}	0.150187	4.09526×10^{-8}	0.151669	6.28066×10^{-8}
0.3	0.227027	8.77935×10^{-9}	0.229219	1.11017×10^{-8}	0.231291	1.73203×10^{-8}
0.4	0.310335	2.80348×10^{-9}	0.312965	3.36487×10^{-9}	0.315453	5.11758×10^{-9}
0.5	0.400138	9.20462×10^{-10}	0.402978	4.08603×10^{-10}	0.405665	7.01896×10^{-12}
0.6	0.498003	7.62562×10^{-11}	0.500792	2.06073×10^{-9}	0.503432	5.09238×10^{-9}
0.7	0.605459	4.59149×10^{-10}	0.607922	7.27692×10^{-9}	0.610254	1.72149×10^{-8}
0.8	0.723994	1.12162×10^{-10}	0.72586	2.56535×10^{-8}	0.727628	6.2306×10^{-8}
0.9	0.855046	3.4671×10^{-9}	0.856073	1.02751×10^{-7}	0.857047	2.53755×10^{-7}
1.0	1.	1.04217×10^{-6}	1.	1.50015×10^{-6}	1.	4.82583×10^{-6}

doi:10.1371/journal.pone.0117368.t005

Table 6. RK4 solutions along with absolute residuals for various γ when *M* = 1 and *R* = 0.3

ξ	γ = 0.5		γ = 0.7		γ = 1.0	
	Solution	Residual	Solution	Residual	Solution	Residual
0.0	0.	2.48394×10^{-4}	0.	3.21426×10^{-5}	0.	5.98185×10^{-6}
0.1	-0.00104157	1.30619×10^{-5}	0.0551344	1.7383×10^{-6}	0.0754476	3.25803×10^{-7}
0.2	0.00408732	3.20449×10^{-6}	0.11299	4.27107×10^{-7}	0.152381	8.00859×10^{-8}
0.3	0.0215448	8.82603×10^{-7}	0.176287	1.18155×10^{-7}	0.232285	2.21753×10^{-8}
0.4	0.0574657	2.56677×10^{-7}	0.247744	3.46488×10^{-8}	0.316647	6.51069×10^{-9}
0.5	0.117953	1.1241×10^{-8}	0.330078	1.03934×10^{-9}	0.406955	1.84115×10^{-10}
0.6	0.209071	2.90569×10^{-7}	0.426004	3.78674×10^{-8}	0.5047	7.08189×10^{-9}
0.7	0.336849	9.76162×10^{-7}	0.538238	1.27274×10^{-7}	0.611375	2.37814×10^{-8}
0.8	0.507281	3.57223×10^{-6}	0.669498	4.63136×10^{-7}	0.728478	8.64159×10^{-8}
0.9	0.726341	1.46036×10^{-5}	0.822506	1.8911×10^{-6}	0.857515	3.52838×10^{-7}
1.0	1.	3.09347×10^{-4}	1.	3.76646×10^{-5}	1.	6.92864×10^{-6}

doi:10.1371/journal.pone.0117368.t006

Table 7. Different order solutions along with absolute residuals when *R* = 1, γ = 1 and *M* = 3.

ξ	First Order		Third order		Fifth order	
	Solution	Residual	Solution	Solution	Residual	Solution
0.0	0.	0.	0.	0.	0.	0.
0.1	0.0746781	2.09932×10^{-3}	0.0746634	3.0637×10^{-6}	0.0746634	2.25321×10^{-9}
0.2	0.0746781	3.77846×10^{-3}	0.150878	4.79033×10^{-6}	0.150878	2.65694×10^{-9}
0.3	0.23023	4.70572×10^{-3}	0.230192	4.54961×10^{-6}	0.230192	9.23882×10^{-10}
0.4	0.31419	4.71352×10^{-3}	0.314146	2.7082×10^{-6}	0.314146	1.56557×10^{-9}
0.5	0.404314	3.84864×10^{-3}	0.404268	3.42113×10^{-7}	0.404268	3.09459×10^{-9}
0.6	0.502119	2.38449×10^{-3}	0.502076	1.42988×10^{-6}	0.502076	2.99595×10^{-9}
0.7	0.609108	7.83349×10^{-4}	0.609071	2.05266×10^{-6}	0.609071	1.86451×10^{-9}
0.8	0.72677	4.0313×10^{-4}	0.726742	1.6919×10^{-6}	0.726742	7.09482×10^{-10}
0.9	0.856579	7.06734×10^{-4}	0.856564	8.84155×10^{-7}	0.856564	1.02005×10^{-10}
1.0	1.	0.	1.	0.	1.	8.59397×10^{-21}

doi:10.1371/journal.pone.0117368.t007

Fig. 5 shows the effect of constant containing permeability M on the velocity profiles. In these profiles, we varied M as $M = 1,3,6,9$, and find that the normal velocity increases with the increase in M while longitudinal velocity increases near the central axis of the channel and decreases near the wall.

The effect of γ on the velocity profiles is depicted in Fig. 6. In these profiles we varied γ as $\gamma = 0.8,1,1.5,3$ and noted that normal velocity increases with the increase in γ whereas longitudinal velocity increases near the central axis of the channel and decreases near the plates.

The effect of $R = M$ on the velocity profile is given in Fig. 7. In these profiles, we see that the normal velocity decreases with the increase in $R = M$ while longitudinal velocity increases near the wall and decreases near the central axis of the channel.

S1, S2 and S3 Figs. depict the effects of $M = \gamma$, $R = \gamma$ *and* $R = M = \gamma$ on the velocity profiles respectively. In these profiles, we observed that normal velocity increases with the increase in $M = \gamma$, $R = \gamma$ *and* $R = M = \gamma$ respectively while longitudinal velocity decreases near the wall and increases near the central axis of the channel.

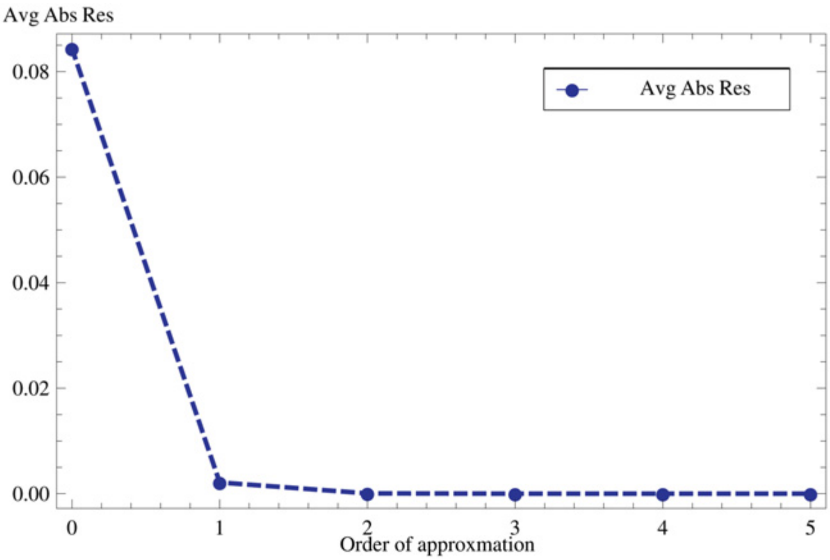

Fig 2. Convergence of HPM solution. Different order solutions along with absolute residuals shows the convergence of the HPM Solution.

doi:10.1371/journal.pone.0117368.g002

It can be observed from these profiles that similar behavior of normal and longitudinal velocity has been captured when we vary $M,\gamma,R = \gamma,M = \gamma$ *and* $R = M = \gamma$ while keeping other parameters fixed. It is also observed that R and $R = M$ have a similar effect on the normal and longitudinal velocity profiles while keeping other parameters fixed.

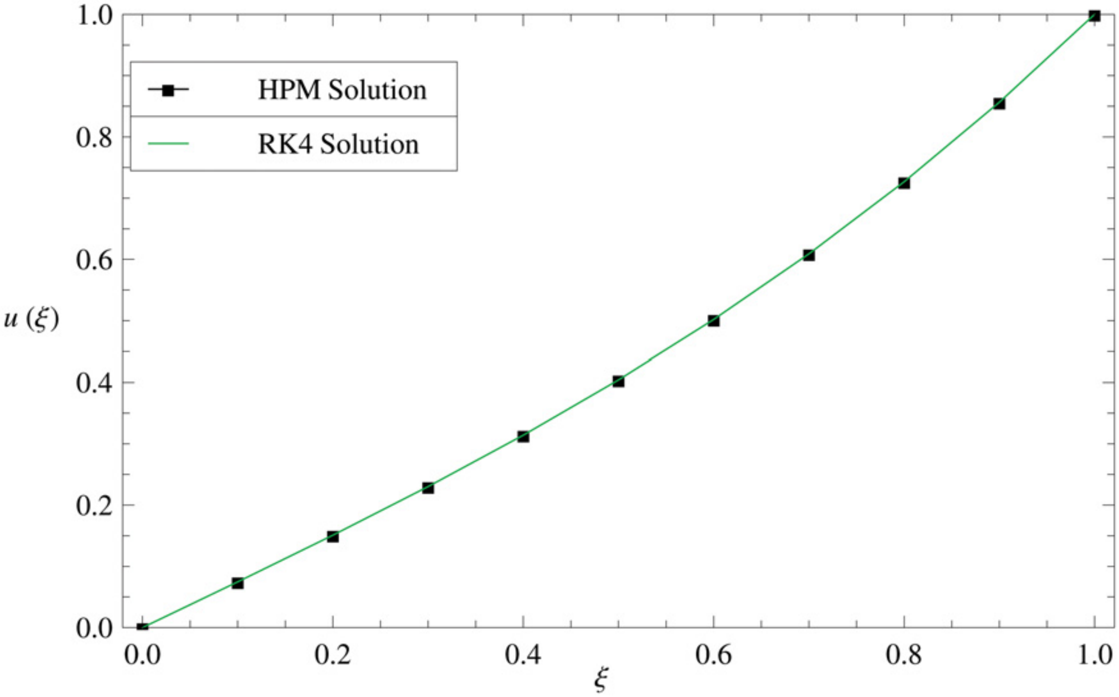

Fig 3. Comparison of HPM and RK4 solutions. Comparison of analytical and numerical solutions shows the validity of HPM solutions.

doi:10.1371/journal.pone.0117368.g003

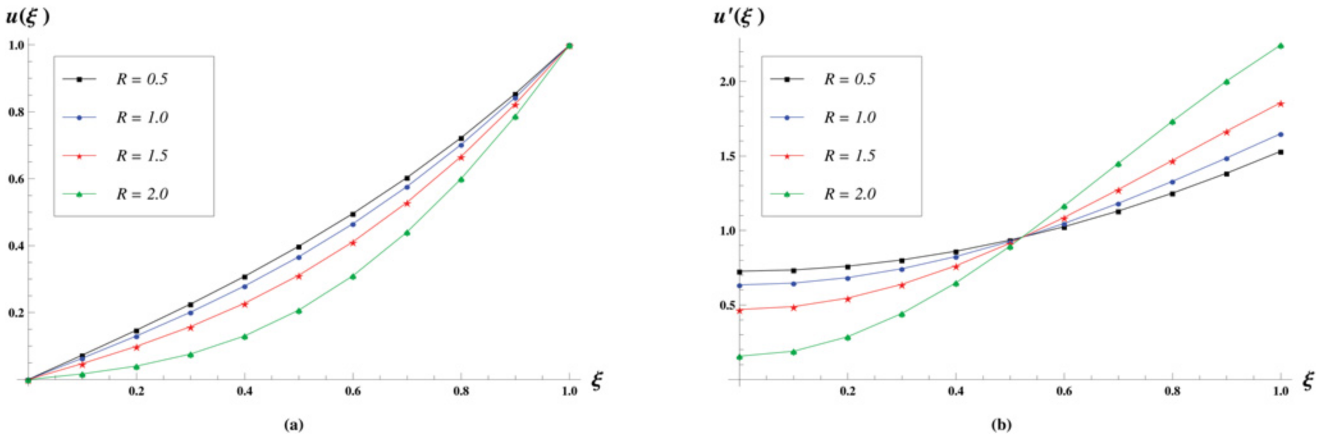

Fig 4. Velocity profiles for various values of *R* = 0.5,1,1.5,2 keeping *M* = 1 and γ = 1 fixed. The effect of Reynolds number *R* on the Normal velocity profiles is shown in (a) while the effect on the longitudinal velocity profiles is shown in (b).

doi:10.1371/journal.pone.0117368.g004

Conclusions

In this article, we find the similarity solution for an unsteady axisymmetric squeezing flow of incompressible Newtonian fluid through porous medium with slip boundary condition using HPM analytically and RK4 numerically. We determined the convergence of HPM solution using various order approximate solutions. In addition, we checked the validity of HPM solution by comparing analytical and numerical solutions. We observed some key findings related to the effects of dimensionless parameters on the velocity profiles. It was found that:

- The normal velocity decreases with the increase in Reynolds number *R*.
- With the increase in Reynolds number *R*, longitudinal velocity increases near the walls and decreases near the central axis of the channel.
- The normal velocity monotonically increases and the longitudinal velocity monotonically decreases from $\xi = 0$ to $\xi = 1$ for fixed positive value of *R* at any given time.

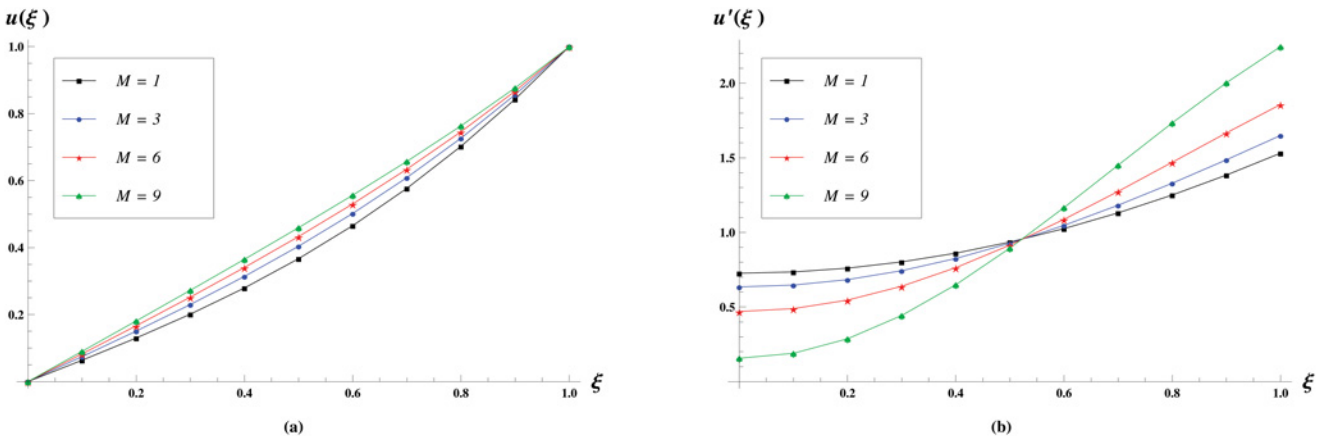

Fig 5. Velocity profiles for various values of *M* = 1,3,6,9 keeping *R* = 1 and γ = 1 fixed. The effect of permeability constant *M* on the Normal velocity profiles is shown in (a) while the effect on the longitudinal velocity profiles is shown in (b).

doi:10.1371/journal.pone.0117368.g005

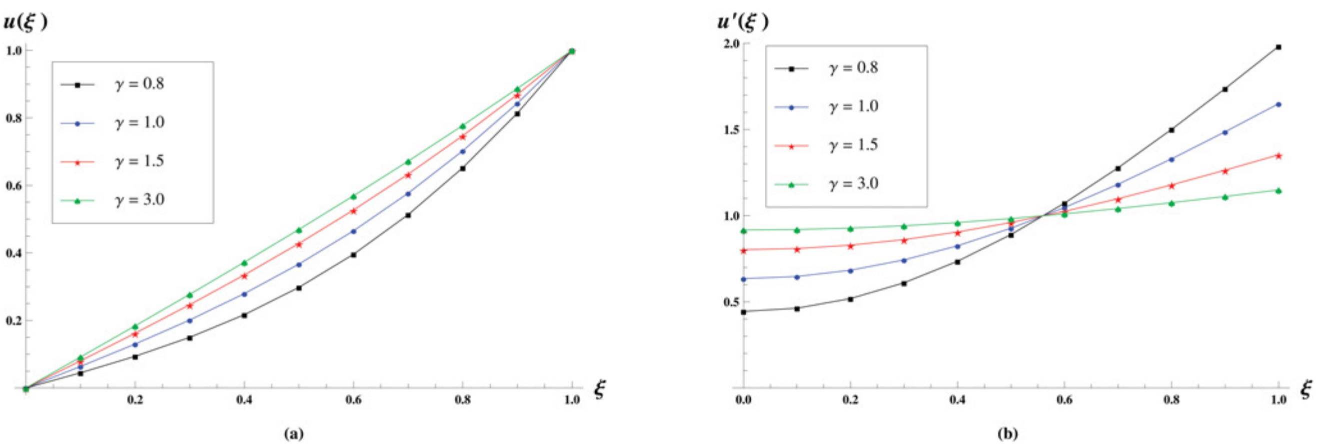

Fig 6. Velocity profiles for various values of *γ* = 0.8,1,1.5,3 keeping *R* = 1 and *M* = 1 fixed. The effect of slip parameter *γ* on the Normal velocity profiles is shown in (a) while the effect on the longitudinal velocity profiles is shown in (b).

doi:10.1371/journal.pone.0117368.g006

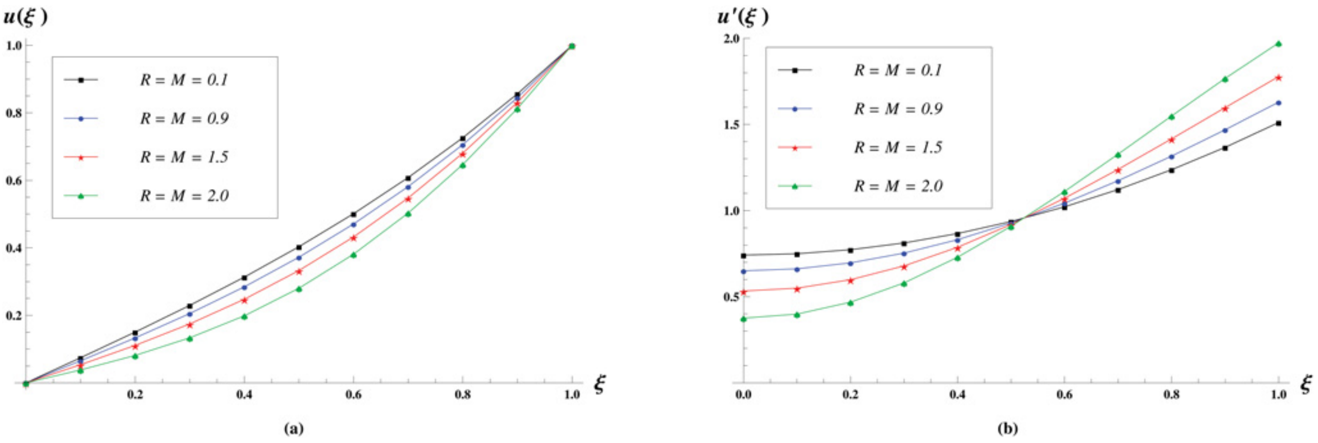

Fig 7. Velocity profiles for various values of *R* = *M* = 0.1,0.9,1.5,2 keeping *γ* = 1 fixed. The effect of Reynolds number *R* = *M* on the Normal velocity profiles is shown in (a) while the effect on the longitudinal velocity profiles is shown in (b).

doi:10.1371/journal.pone.0117368.g007

- R and M have opposite effects, while γ and M have similar effects on the normal and longitudinal velocity components.
- Similar velocity profiles are obtained when we vary $M,\gamma,R = \gamma,M = \gamma$ *and* $R = M = \gamma$, while keeping the remaining parameters fixed.
- R and $R = M$ have a similar effect on the velocity profiles, while keeping the remaining parameters fixed.

Supporting Information

S1 Fig. Velocity profiles for various values of $\gamma = M = 0.8,1.2,1.5,2$ keeping $R = 1$ fixed. The effect of $\gamma = M$ on the Normal and longitudinal component of velocity profile is given in (a) and (b) respectively.
(EPS)

S2 Fig. Velocity profiles for various values of $R = \gamma = 0.6,0.9,1.2,2$ keeping $M = 3$ fixed. The effect of $R = \gamma$ on the Normal and longitudinal component of velocity profile is given in (a) and (b) respectively.
(EPS)

S3 Fig. Velocity profiles for various values of $R = M = \gamma = 0.7,1,1.2,2$. The effect of $R = M = \gamma$ on the Normal and longitudinal component of velocity profile is given in (a) and (b) respectively.
(EPS)

S1 Table. Comparison of HPM and RK4 solutions for various R when $\gamma = 1$ and $M = 3$.
(DOCX)

S2 Table. Comparison of HPM and RK4 solutions for various M when $\gamma = 1$ and $R = 03$.
(DOCX)

S3 Table. Comparison of HPM and RK4 solutions for various γ when $M = 1$ and $R = 0.3$.
(DOCX)

Author Contributions

Conceived and designed the experiments: MQ. Performed the experiments: MQ. Analyzed the data: MQ IU. Contributed reagents/materials/analysis tools: MQ HK MTR. Wrote the paper: MQ IU HK. Problem Modeling: MQ. Review: HK MTR.

References

1. Tan W, Masuoka T (2005) Stokes first problem for a second grade fluid in a porous half-space with heated boundary. International Journal of Non-Linear Mechanics 40: 515–522.
2. Hamdan MH (1998) An alternative approach to exact solutions of a special class of Navier-Stokes flows. Applied Mathematics and Computation 93: 83–90.
3. Hamdan MH, Allan FM (2006) A note on the generalized Beltrami flow through porous media. International Journal of Pure and Applied Mathematics 27: 491–500.
4. Vafai K, Tien CL (1981) Boundary and inertia effects on flow and heat transfer in porous media. International Journal of Heat and Mass Transfer 24: 195–203.
5. Islam S, Mohyuddin MR, Zhou CY (2008) Few exact solutions of non-Newtonian fluid in porous medium with hall effect. Journal of Porous Media 11: 669–680.
6. Islam S, Zhou CY (2007) Certain inverse solutions of a second-grade magneto hydrodynamic aligned fluid flow in a porous medium. Journal of Porous Media 10:401–408.
7. Vafai K (2005) Hand Book of Porous Media. CRC Press, Taylor & Francis Group.
8. Stefan MJ (1874) Versuch Über die scheinbare adhesion. Sitzungsberichte/Österreichische Akademie der Wissenschaften in Wien Mathematisch-Naturwissenschaftliche Klasse 69: 713–721. PMID: 4920446
9. Thorpe JF, Shaw WA (1967) Developments in Theoretical and Applied Mechanics. Pergamon Press, Oxford.
10. Gupta PS, Gupta AS (1977) Squeezing flow between parallel plates. Wear 45: 177–185.
11. Kuzma DC (1968) Fluid inertia effects in squeeze films. Applied Scientific Research 18: 15–20.
12. Elkouh AF (1986) Fluid inertia effects in squeeze film between two plane annuli. Journal of Tribology 106: 223–227.
13. Verma RL (1981) A numerical solution for squeezing flow between parallel channels. Wear 72: 89–95.
14. Singh P, Radhakrishnan V, Narayan KA (1990) Squeezing flow between parallel plates. Ingénieur-Archives 60: 274–281.
15. Leider PJ, Bird RB (1973) Squeezing flow between parallel disks. I. Theoretical analysis, Industrial & Engineering Chemistry Fundamentals 13: 336–341.

16. Naduvinamani NB, Hiremath PS, Gurubasavaraj G (2001) Squeeze film lubrication of a short porous journal bearing with couple stress fluids. Tribology International 34: 739–747.
17. Islam S, Khan H, Shah IA, Zaman G (2011) Anaxisymmetric squeezing fluid flow between the two infinite parallel plates in a porous medium channel. Mathematical Problems in Engineering.
18. Hamza EA (1988) The Magnetohydrodynamic squeeze film. Journal of Tribology 110: 375–377.
19. Domairry G, Aziz A (2009) Approximate analysis of MHD Squeeze flow between two parallel disks with suction or injection by homotopy perturbation method. Mathematical Problem in Engineering.
20. Qayyum A, Awais M, Alsaedi A, Hayat T (2012) Flow of Jeffery Fluid between Two Parallel Disks. Chinees Physics Letters 29: 034701.
21. Grimm RJ (1976) Squeezing flows of Newtonian liquid films an analysis include the fluid inertia. Applied Scientific Research 32(2): 149–166.
22. Tichy JA, Winer WO (1970) Inertial considerations in parallel circular squeeze film bearings. Journal of Lubrication Technology 92: 588–592.
23. Laun HM, Rady M, Hassager O (1999) Analytical solutions for squeeze flow with partial wall slip. Journal of Non-Newtonian Fluid Mechanics 81:1–15.
24. Ishizawa S (1966) The unsteady flow between two parallel discs with arbitrary varying gap width. Bulletin of the Japan Society of Mechanical Engineers 9: 533–550.
25. Neto C, Evans DR, Bonaccurso E, Butt HJ, Craig VSJ (2005) Boundary slip in Newtonian liquids: a review of experimental studies. Reports on Progress in Physic 68: 2859.
26. Navier CLMH (1823) Mémoire sur les lois du mouvement des fluides. Mémoires de l'Académie Royale des Sciences de l'Institut de France 6: 389–440.
27. He JH (1999) Homotopy perturbation technique. Computer Methods in Applied Mechanics and Engineering 178: 57–262.
28. He JH (2000) A coupling method of homotopy technique and perturbation technique for nonlinear problems. International Journal of Non-linear Mechanics 35: 37–43.
29. He JH (2003) Homotopy perturbation method, a new non-linear analytical technique Applied Mathematics and Computation 135: 73–79.
30. He JH (2006) Homotopy perturbation method for solving boundary value problems Physics Letters A 350:87–88.
31. Kolyshkin A, Nazarovs S (2007) Stability of slowly diverging flows in shallow water. Mathematical Modeling and Analysis 12(1): 101–106.
32. Krylovas A, Ciegis R (2001) Asymptotical analysis of one dimensional gas dynamics equations. Mathematical Modeling and Analysis 6(1): 117–128.
33. Nayfeh AH (1981) Introduction to Perturbation Techniques. John Wiley & Sons, New York; Chichester.
34. He JH (2006) New interpretation of homotopy perturbation method. International Journal of Modern Physics B, 20(18): 2561–2568.
35. Siddiqui AM, Ahmed M, Ghori QK (2006) Couette and Poiseuille flow for Non Newtonian fluids. International Journal of Nonlinear Sciences and Numerical Simulation, 7(1):15–26.
36. Siddiqui AM, Mahmood R, Ghori QK (2006) Thin film flow of a third grade on a moving belt by He's Homotopy Perturbation method. International Journal of Nonlinear Sciences and Numerical Simulation 7(1): 7–14.
37. Zhou G, Wu B (2014) Application of the homotopy perturbation method to an inverse heat problem. International Journal of Numerical Methods for Heat & Fluid Flow 24(6): 1331–1337.
38. Khan H, Islam S, Ali J, Shah IA (2012) Comparison of different analytic solutions to axisymmetric squeezing fluid flow between two infinite parallel plates with slip boundary conditions. Abstract and Applied Analysis.
39. Breugem WP (2007) The effective viscosity of a channel-type porous medium. Physics of Fluids 19(10). PMID: 19816550
